# Addressing challenges in tuberculosis adherence via performance-based payments for integrated case management: protocol for a cluster randomized controlled trial in Georgia

**DOI:** 10.1186/s13063-019-3621-z

**Published:** 2019-08-28

**Authors:** Ivdity Chikovani, Karin Diaconu, Predrag Duric, Lela Sulaberidze, Maia Uchaneishvili, Nuredin Ibrahim Mohammed, Akaki Zoidze, Sophie Witter

**Affiliations:** 1 Curatio International Foundation, 3 Kavsadze Str., 0179 Tbilisi, Georgia; 2 Queen Margaret University, Edinburgh, UK; 3 MRC Unit The Gambia at the London School of Hygiene & Tropical Medicine, Banjul, The Gambia

**Keywords:** Tuberculosis, Performance-based financing, Integrated care, Adherence, Loss to follow-up, Cluster randomized trial

## Abstract

**Background:**

Tuberculosis is one of the greatest global health concerns and disease management is challenging particularly in low- and middle-income countries. Despite improvements in addressing this epidemic in Georgia, tuberculosis remains a significant public health concern due to sub-optimal patient management. Low remuneration for specialists, limited private-sector interest in provision of infectious disease care and incomplete integration in primary care are at the core of this problem.

**Methods:**

This protocol sets out the methods of a two-arm cluster randomized control trial which aims to generate evidence on the effectiveness of a performance-based financing and integrated care intervention on tuberculosis loss to follow-up and treatment adherence.

The trial will be implemented in health facilities (clusters) under-performing in tuberculosis management. Eligible and consenting facilities will be randomly assigned to either intervention or control (standard care). Health providers within intervention sites will form a case management team and be trained in the delivery of integrated tuberculosis care; performance-related payments based on monthly records of patients adhering to treatment and quality of care assessments will be disbursed to health providers in these facilities.

The primary outcomes include loss to follow-up among adult pulmonary drug-sensitive and drug-resistant tuberculosis patients. Secondary outcomes are adherence to treatment among drug-sensitive and drug-resistant tuberculosis patients and treatment success among drug-sensitive tuberculosis patients. Data on socio-demographic characteristics, tuberculosis diagnosis and treatment regimen will also be collected. The required sample size to detect a 6% reduction in loss to follow-up among drug-sensitive tuberculosis patients and a 20% reduction in loss to follow-up among drug-resistant tuberculosis patients is 948 and 136 patients, respectively.

**Discussion:**

The trial contributes to a limited body of rigorous evidence and literature on the effectiveness of supply-side performance-based financing interventions on tuberculosis patient outcomes. Realist and health economic evaluations will be conducted in parallel with the trial, and associated composite findings will serve as a resource for the Georgian and wider regional Ministries of Health in relation to future tuberculosis and wider health policies.

The trial and complementing evaluations are part of Results4TB, a multidisciplinary collaboration engaging researchers and Georgian policy and practice stakeholders in the design and evaluation of a context-sensitive tuberculosis management intervention.

**Trial registration:**

ISRCTN, ISRCTN14667607. Registered on 14 January 2019.

**Electronic supplementary material:**

The online version of this article (10.1186/s13063-019-3621-z) contains supplementary material, which is available to authorized users.

## Background

Tuberculosis (TB) is one of the greatest global public health challenges: in 2017 alone, 10 million people developed the disease, with over 1.5 million of these cases resulting in death [1]. While technological and medical advances are increasingly assisting in disease detection [2] and treatment [3], TB management still presents substantial challenges, particularly in low- and middle-income countries (LMICs) [1]. One of the targets of the Sustainable Development Goals for 2030 is to end the global TB epidemic. The 2014 World Health Organization (WHO) “End TB Strategy” calls for a 90% reduction in TB deaths and an 80% reduction in TB incidence over 2015–2030 [4]. Countries must progress quickly to prevent, detect and treat TB if these targets are to be achieved.

### Country setting

Countries in Eastern Europe and Central Asia face particularly high burdens of TB, including multidrug-resistant TB (MDR-TB) [1]. Increased drug resistance, low rates of case detection and treatment adherence, as well as system-level issues such as funding gaps and limited integration of TB services across provider types (public and private, secondary and primary care, and patient communities), all pose critical challenges for TB management [1]. Contextually sensitive approaches are urgently needed to address such challenges and improve TB prevention, detection and treatment.

The current study focuses on Georgia, a country challenged by a particularly high TB burden. Latest estimates record TB incidence at 86 (range 72–101) per 100,000 persons, the largest proportion of which (80%) is made up of patients with pulmonary TB [5]. The majority of patients with TB is comprised of patients with drug-susceptible TB (DS-TB; approximately 88.1%) [6]; in 2016, the estimated prevalence of rifampicin-resistant MDR-TB (MDR-RR-TB) was 11% among new patients with TB and 30% among previously treated patients [5].

In line with global averages, overall treatment success was estimated at 84% for both new and relapsed TB (2015 estimate). However, the rate fluctuates widely across regions of Georgia and reporting health facilities: for DS-TB, treatment success ranges from 46 to 100% (2015 estimates) [6]; and for MDR-RR-TB, estimates from 2015 place treatment success at 56% [5].

In both cases, low treatment success is attributed to weaknesses in patient management [7], which result in poor patient adherence and patients being lost to follow-up (LFU) (approximately 30%) [8]. At the core of this problem is limited provider motivation to provide tuberculosis services given the infectious nature of the disease and low remuneration received by specialists. The latter additionally presents problems for retaining and attracting staff towards tuberculosis service delivery. Reducing the LFU rate is not only essential to ensure improved patient outcomes, but also to prevent the spread of MDR-TB. Promising interventions in this regard include integrated care management and performance-based financing [9].

### TB care in Georgia

An extensive overview of the current systems of care for TB management in Georgia is available online [10]; we summarize most salient points as follows. TB services in Georgia are part of general health care services, which underwent significant reorganization after a recent privatization. The provision of health care is now based on a public–private mix, where the government finances public programmes and health services (e.g. for TB or diabetes) delivered by private providers. At present, the government supports universal free access to TB care, although the financing of TB service provision depends in part on funding from the Global Fund (GF).

At the outpatient level, TB services are provided at TB units by TB doctors and TB nurses. There are currently 68 TB units in the country: 58 semi-urban (located in district centres) and 10 urban TB units. Abkhazia and South Ossetia are out of control territories and therefore the state health programmes are not provided to the population residing in these territories.

Semi-urban units were recently integrated into district and regional level Primary Health Care (PHC) centres, most of which are private. Only a few TB units remain as separate public institutions, mainly in the capital and main cities of the country (10 urban centres). The latter units serve a greater number of patients in total compared to the accumulated number of patients receiving services in private outpatient units.

Outpatient treatment is provided by TB units located in urban and semi-urban areas and follows the direct observation therapy (DOT) strategy. TB units are staffed by TB doctors and DOT nurses. There is no difference in service provision at TB units integrated into PHC (mostly private facilities in semi-urban areas) and in TB units administratively not integrated into PHC, which are in most cases public TB facilities in urban areas. In rural areas, DOT is provided by a general primary care nurse under the supervision of the TB doctor.

*Integrated care management*, whereby diverse and multidisciplinary service providers collaborate to offer patient-centred services, is an increasingly popular policy for complex disease management. This is likely to be of particular value for TB, where management at the primary care level is based on a variation of the traditional “cascade of care” approach, whereby a TB specialist works together with general practitioners, managing the condition in a coordinated and bidirectional manner [11]. In such set-ups, specialists retain responsibility for clinical management and treatment oversight, while primary care providers perform additional supportive functions. The latter can range from improving patient follow-up at the PHC level (as in Norway [12] and Canada [13]) to direct case management by general practitioners (as in Romania [14], Turkey [15, 16] and India [17]).

Due to various social determinants underlying TB onset and the complex needs of patients, successful TB management requires the input of multiple disciplines. Several countries, including the United Kingdom and Norway, have achieved relatively successful TB management via multidisciplinary teams (MDTs) which provide comprehensive and patient-centred care [11]. MDTs usually consist of professionals with a mix of skills to meet the needs of patients with TB, especially those with more complex MDR-TB who require additional psycho-emotional and clinical support [11].

Each country still has its challenges in fighting TB, yet international evidence shows that well-designed and integrated models of primary care engagement can improve treatment access to TB treatment, increase case notification and improve treatment adherence and success [11]. However, successful service integration around TB control is predicated on wider health system factors and performance, including availability and specialized training of human resources, managerial competence and interest as relates to TB service oversight as well as financial viability of service delivery.

*Performance-based financing* (or results-based financing) schemes are interventions where a cash payment or non-monetary transfer is made conditional upon attainment and verification of predefined service outputs or quality [18]. While prevalent internationally, a 2012 Cochrane review on performance-based financing (PBF) schemes across low- and middle-income countries suggested that evidence on the effectiveness of PBF on health service delivery was weak [19]. Some positive effects on outputs had been shown in some settings, but the number and quality of evaluations on the topic was poor and PBF is a highly heterogeneous intervention, and highly context and implementation dependent [19]. This suggests clear potential for the proposed clinical trial to add to the research and evidence base on this topic.

A more recent review of the effectiveness of supply-side PBF interventions on TB service delivery across low-, middle- and high-income countries reports similar findings [20]. Current research suggests that PBF schemes may impact positively on case detection, service utilization as well as treatment success. Regarding the latter, research from Europe (Czech Republic and Romania) suggests that cure rates may improve by over 20% in settings applying PBF [21]; indeed, patients treated in centres operating under PBF schemes are found to be at increased odds of treatment success. However, despite promising results, the studies appraised are highly heterogeneous and the evidence base is mixed. Similar to Witter et al.’s [19] study, RCT evidence is sparse and available studies suggest that intervention success is highly dependent upon the scheme design, operationalization and fit to context. Careful calibration of financial incentives to national and regional contexts is one of the factors affecting PBF scheme success [20].

Two further issues are of relevance to PBF operationalization, particularly as linked to integrated care management [20]:

- Schemes encouraging care coordination show promise; however, clear links between payments and desired functions must be drawn. Evidence from Taiwan suggests that establishing multidisciplinary teams and assigning overall coordination responsibilities to one professional cadre may improve TB management. However, care must be taken to specify and communicate how rewards are linked to specific coordination (or other PBF) functions so as to ensure prompt translation into clinical practice.
- PBF schemes and incentives must fit social and professional norms. Evidence from China suggests that, if perceived as compromising “professional dignity” and social norms, financial incentives may be sabotaged by health care professionals.

Results4TB is a multidisciplinary research project aiming to engage local Georgian stakeholders in an evidence-based intervention co-design process prior to rigorous intervention evaluation via randomized trial (this protocol) and health economic and realist evaluation [22]. Driven by policy interests, researchers conducted rapid evidence reviews of performance-based financing interventions and additionally convened two workshops with health care professionals active in TB care to explore issues affecting TB service delivery and the cascade of care. Workshops confirmed that weak remuneration and limited provider motivation were issues to contend with in the Georgian contexts; however, further issues contributing to high loss to follow-up rates were raised, including the lack of communication between providers and a lack of service integration. Via repeat contact with policy-makers, and drawing on previous work by the research team on integrated care, a comprehensive performance-based financing intervention promoting integrated TB service delivery was developed and will be evaluated by the research team [9].

This protocol describes the methods of a two-arm cluster randomized control trial that seeks to establish the effectiveness of the aforementioned intervention against standard care. The trial is conducted in parallel with a realist and health economic evaluation, and thus offers a unique opportunity to study how and why implementation of care integration and performance-based financing is operationalized in Georgia while simultaneously studying the cost-effectiveness of such an intervention.

## Methods

### Aims

This trial aims to determine the effect of an integrated care and performance-based financing intervention on loss to follow-up and patient adherence to treatment among adult patients with pulmonary drug-susceptible and drug-resistant TB in Georgia.

### Objectives

The primary objective of the trial is to compare loss to follow-up among both drug-susceptible and drug-resistant pulmonary TB patients between intervention and control arms. Secondary objectives include comparison of adherence to treatment among patients with pulmonary DS-TB and patients with pulmonary DR-TB in the two trial arms measured at the end of each completed treatment month for a patient. Additional secondary objectives include comparison of treatment success rates among patients with pulmonary DS-TB in the two trial arms.

### Study design

This is a pragmatic, cluster randomized controlled, superiority trial with two parallel groups, a fixed number of clusters of unequal size and a primary outcome of loss to follow-up among drug-sensitive and drug-resistant TB outpatients. A clustered design—featuring health facilities as clusters—was chosen as facilities represent the ideal frame to disburse performance-related payments to TB providers. Randomization will be constrained and stratified according to the facility operation type (i.e. integrated vs. specialized care management) and clusters will be allocated to intervention and control arms on a 1:1 ratio. Via constrained randomization we will assure no substantive baseline imbalance exists among clusters in relation to patient load (values from 2016 and 2017), treatment-related loss to follow-up and success among drug-susceptible patients (values from 2016), and the number of human resources (doctors and nurses) and their average salary***.***

### Study setting

This study will be carried out in private and public health facilities in Georgia meeting the following cluster (health facility) eligibility criteria. A full list of eligible facilities is presented in Appendix 1.

### Cluster eligibility

To be eligible for the trial, health facilities must comply with all the following criteria at randomization:

- Facilities must include a TB unit (TB unit at a specialized TB care facility, or TB unit integrated into primary health care).
- Facilities must have registered at least 20 patients with pulmonary DS-TB as treated in the baseline year used for sampling purposes (2016).
- Facilities must have a pulmonary DS-TB treatment success rate of 82% or less in the baseline year.
- Facilities must have at least one TB doctor and one TB nurse available within the facility.
- Managers of health care facility networks, managers of a single private health care facility providing general outpatient care or managers of specialized facilities must consent and agree to participate in the trial, including agreement to disburse performance-related payments as set out in the intervention.

Clusters will be excluded if they are taking part in any other clinical trials on treatment regimen, new drugs and adherence to treatment, and/or if outcome monitoring frameworks of the facility are assessed as weak (and therefore inflexible for intervention) by the National TB Directory.

We will not exclude clusters in the case of implementation of interventions that do not fall into the stated category (e.g. video observed therapy, concomitant chronic disease care, HIV care, acute disease care).

### Patient eligibility

To be eligible for participation in the trial, patients must be:

- Aged 18 years or over
- A new or previously treated patient diagnosed bacteriologically or clinically with pulmonary TB (PTB); diagnosis must have been established by a direct sputum smear microscopy, culture or Gene Xpert MTB/RIF (bacteriologically confirmed case), or X-ray, histological or morphological changes (clinically diagnosed case)
- Assigned to outpatient TB treatment not more than 1 month prior to enrolment in the study; this restriction (not more than 1 month of treatment) does not refer to patients whose most recent treatment outcome was a failure and who were assigned to a new treatment regimen
- Assigned to outpatient TB treatment at an outpatient facility or at home (through DOT or video observed therapy (VOT)) according to the national TB treatment guidelines.

Patients will be excluded if:

- Diagnosis is extrapulmonary TB
- TB outpatient treatment was started more than 1 month prior to enrolment in the study
- Patients with DS-TB have undergone more than 2 months of hospitalization or patients with DR-TB have undergone more than 6 months of hospitalization
- Patients are known at the start of treatment to require treatment longer than is recommended by the Georgian TB Management Guidelines for the appropriate type of TB (Appendix 2)
- Patients are custody patients
- Patients are involved in other clinical studies related to TB treatment
- Patients are leaving the area within the next 6 months.

### Provider eligibility

All facility managers and health care providers offering TB treatment in eligible facilities are eligible to take part in this trial.

### Comparators

The trial is restricted to two arms: intervention and control. In the intervention arm, an integrated care and performance-based financing intervention will be rolled out and implemented by the Ministry of Labour, Health and Social Affairs (MoLHSA) of Georgia and the Social Service Agency across a random group of public and private health care facilities. Patients presenting at the said health facilities will be offered the option to enrol in this study. The control group will comprise health facilities (i.e. clusters) not implementing the intervention and managing TB as per standard treatment protocols and processes.

### Explanation for choice of comparators

While until now no facility or individual-level provider performance incentive schemes have been implemented for primary or secondary care services in Georgia (including TB and other services), there is now considerable interest in performance-based financing schemes to strengthen the quality of services generally, and TB care specifically. The intervention under study has been designed in collaboration with Georgian policy and practice actors [22], and as such directly reflects current policy and practice priorities and interests. Given the limited information on likely intervention effectiveness, standard care was chosen as the control.

*The intervention package* includes results-based financing of health care providers and delivery of integrated care at the primary care (outpatient level) with the aim to reduce loss to follow-up among pulmonary DS-TB and DR-TB patients and to improve TB treatment success in patients with pulmonary DS-TB. The intervention aims to create a better relationship between a patient with TB and providers by adopting a patient-centred care approach. The intervention theory of change views this as a first step in promoting patient engagement in the decision-making process, thus resulting in improved trust and improved management of co-morbidities and side effects of TB medication, which in turn contribute to better adherence to treatment. Performance-based payments are made towards the TB case management team based on quarterly reports summarizing monthly adherence to treatment among pulmonary TB patients. The bonus payments to a TB unit considers the number of patients with TB; cascading of payment to team members is based on their estimated contribution towards the care process. Payments to team members have additionally been calibrated against basic salaries to ensure the relative magnitudes of bonus payments are appropriate.

Two intervention models are proposed considering the current set-up of TB services at the outpatient level in Georgia:

- Intervention model 1 for TB units administratively integrated into primary care facilities
  This intervention model applies principally to health facilities located in semi-urban areas of Georgia. TB patients which present for care in these intervention facilities will receive treatment from an integrated, multidisciplinary team; the care team consists of a TB doctor, a TB nurse (or a rural nurse for rural patients) and a family doctor (or a rural doctor for rural patients); the facility manager is an additional member of the wider TB service management team.
  For these facilities, roles and responsibilities of health care providers involved in TB case management have been revised (within the scope of existing staff competencies) to ensure that roles align with principles of integrated care management (see Additional file 1). Staff in the newly established multidisciplinary team will additionally receive training on principles of integrated care, and on managing the side effects of TB treatment in particular. The latter is targeted at family and TB doctors.
  New tools (a treatment plan for TB patients, instruments for adherence monitoring, facility manager guideline on intervention implementation) complement the revision in roles and responsibilities and associated trainings. Bonuses to facilities and staff therein are paid on a quarterly basis based on monthly adherence reports and quarterly structured quality of care assessments (see Additional file 1).
- Intervention model 2 for TB units NOT administratively integrated into primary care facilities
  This intervention model will be adopted by facilities which operate specialized TB services; these facilities are typically free-standing hospitals and treatment units in urban areas. In the current model of care for these facilities, TB doctors and TB nurses are principally involved in care provision; the facility manager is an additional member of the wider TB service management team.
  Similar to intervention model 1, the TB doctor and TB nurse team are expected to deliver integrated person-centred care. Training on the management of side effects of TB medications will be provided for TB doctors and new tools supporting the intervention (a case management plan, instruments for monitoring, a facility managers’ guideline on implementing the intervention) will be introduced. Bonuses to facilities and staff therein are paid on a quarterly basis based on monthly adherence reports (see Additional file 1).

In both models, the facility manager plays a vital role in the delivery of the intervention package. The manager will be responsible for enabling the work of the team (such as contracting, creating job descriptions, supervision on the bonus distribution among the team, etc.) and ensuring a supportive environment for providing patient-centred care. The total bonus payment for the facility includes the manager’s and the institution’s share (the latter potentially covering some additional costs linked to the intervention, such as communications). For further details on the intervention, see Additional file 1.

### Control

Facilities assigned to the control arm will continue to provide outpatient care as per standard national TB management guidelines, and treatment follows DOT strategies. For patients with DS-TB, flexible DOT is practised. Patients should visit DOT sites three times per week, in certain cases twice per week, and take their drugs for the next day(s) with them. Patients with DR-TB should present at DOT sites six times per week (Monday–Saturday). VOT is practised in a small number of the patients with DR-TB, although this is likely to expand.

Outpatient treatment is provided by TB units located in urban and semi-urban areas and staffed by TB doctors and DOT nurses. DOT in urban areas is provided by DOT nurses under the supervision of TB doctors. In practice, in large urban areas, TB nurses and patients with DS-TB carry out a modified DOT regimen: the nurse and the patient agree to meet outside the TB unit three times per week. At this point, the nurse delivers the medicines to the patient, but no observation of treatment is done.

In rural areas, primary care nurses are assigned to DOT implementation (for both patients with DR-TB and those with DS-TB). They undertake DOT under the supervision of the TB doctor working at the TB unit at district level but in the same geographic location.

### Allocation

Sixteen units are eligible for participation in the trial. Clusters will be randomly assigned to either the control or experimental group with a 1:1 allocation ratio as per a computer-generated randomization schedule. Units will be stratified according to the type of intervention to be implemented (i.e. stratification into specialized vs. integrated units). Within each stratum, constrained randomization techniques will be used to allocate a set number of units to the intervention and control arms: three specialized and five integrated units will act as intervention sites; and three specialized and five integrated units will act as control sites. In total, eight units will be allocated to the intervention arm and eight to the control arm, respectively. Constrained randomization will be used to generate the allocation schedule and will be carried out by the research team. The technique verifies that no baseline imbalances exist with regards to the following variables: DS-TB and DR-TB cohort sizes in 2016 and 2017, loss to follow-up and success rates in 2016; and TB unit ownership type and number of doctors and nurses and their salaries. Randomization will only take place after all TB units give agreement to participate in the study.

Mitigation strategies and actions have been noted in case of any changes affecting facility clusters during the study period. If TB units in the control group merge during the study, the statistical analysis will account for clustering as appropriate. If a control unit and an intervention unit merge during the study, the intervention will continue for the merged unit; the principal analysis will not include these data, but extended analyses will be run including data as appropriate for time periods pre merger. If the number of all TB patients decreases below 10 during a quarter, extended statistical analyses will also account for this trend. Should a national/regional-level change occur in the salary paid out to health care providers (a 25% increase or more), recalculation of the bonus amount will be considered by the intervention manager in the MoLHSA.

Strategies for improving facility adherence to intervention are foreseen within the intervention itself. The MoLHSA will support the research team to develop operational guidelines for facility managers that will provide guidance on the administration of the intervention, roles of the staff members, reporting requirements and so forth. Training will also be provided to the health personnel and facility managers, along with practical activities where different possible scenarios of intervention implementation will be examined. In addition to this, supervision site visits will be delivered by the MoLHSA representatives, where providers will be supported with tips and advice in intervention sites.

### Blinding

Participating units and those administering the intervention will not be blinded. Employees of the participating units (TB doctors, nurses, family doctors) in the intervention arm will be trained in the intervention and they will be receiving bonuses. Employees of the participating units in the control arm will not receive the training and bonuses, but they will be aware of the intervention in the intervention arm. While the intervention will not be promoted among patients, they might know of the presence or the absence of the intervention in the TB unit where they are treated. Once collated, data will undergo a de-identification process, whereby patient and cluster identifiers are removed. Statistical analysts will have access only to this de-identified dataset; for dissemination of findings, the data will be matched to original clusters and intervention/non-intervention sites.

The *primary outcome* is loss to follow-up among adult drug-sensitive tuberculosis patients. Loss to follow-up is defined as the difference between the two intervention arms in the proportion of patients who did not start treatment after diagnosis or whose treatment was interrupted for 2 consecutive months or more. The outcome will be assessed based on patient data collected every treatment month.

*Secondary outcomes* include the following:

- Loss to follow-up among drug-resistant tuberculosis patients (similarly defined as for drug-sensitive patients) at each month of a treatment
- Adherence to treatment among drug-sensitive tuberculosis patients for every month of treatment. The research team discussed the adherence definition during a meeting with a representative from the National Center for Tuberculosis and Lung Diseases (NCTLD), and consensus was achieved at the end of this meeting. The definition draws on local regulations and relevant evidence in this area [23]. The definition of adherence to DOT for patients with pulmonary DS-TB is as follows:
  - From the start of the treatment to treatment completion, not more than 1 missing day per month if the patient visits a facility 8–12 times during a month to fulfil DOT (DOT is performed 2 or 3 days per week; every other day, the patient receives drugs at home)
  - From the start of the treatment to treatment completion, no single missing day per month if the patient visits a facility four times during a month to fulfil DOT (some patients visit a facility only once a week for DOT and take drugs for home-taking)
- Adherence to treatment among drug-resistant tuberculosis patients. Adherence to DOT for patients with pulmonary DR-TB is defined as not more than 3 missing days per month (DOT is performed 26 days per month—6 days a week except Sunday) from the start of the treatment
- Treatment success among drug-sensitive tuberculosis patients. Treatment success is defined as per the WHO [24]. The treatment success rate will be calculated as the difference in the proportion of patients classed as successfully treated between trial arms at 6, 12 and 24 months.

*Additional outcomes* that we will examine in ancillary studies include the following:

- Case detection at a primary health care centre (family doctor level) (via realist evaluation)
- Horizontal coordination between PHC and specialized TB services (via realist evaluation)
- Costs associated with the management of side effects caused by TB treatment, and of comorbidities (via health economic evaluation)
- Outcomes of managing co-morbidities measured by hospitalization rates due to any severe co-morbid conditions (like diabetes, cerebrovascular conditions, etc)

**Fig. 1:**
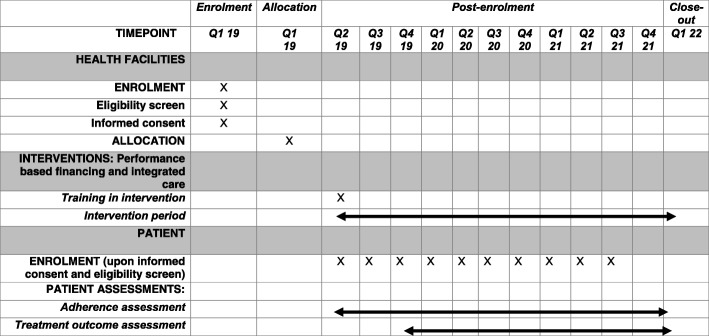
Health facility enrolment and allocation, intervention training and rollout, and patient enrolment and assessment phases

We developed a checklist describing health facility enrolment and allocation, intervention training, rollout, patient enrolment and assessments according to the Standard Protocol Items: Recommendations for Interventional Trials (SPIRIT) guideline (see Fig. 1 and Additional file 2).

## Materials and analysis

### Sample size

Assuming 15% attrition, we estimated that data for 948 pulmonary DS-TB patients and 136 pulmonary DR-TB patients must be included in the trial to detect a 6% reduction in loss to follow-up among DS-TB patients and a 20% reduction in loss to follow-up among DR-TB patients. Sample size calculations assume that all eligible clusters will consent to participate in the trial; calculations were informed by historical data (DS-TB data and DR-TB data from 2016) specific to the 16 eligible clusters. Calculations proceeded in line with recommendations for sample estimation for cluster randomized control trials including a fixed number of clusters of unequal size [25]. We calculated the sample size corresponding to 80% power and 5% significance, accounting for an intra-cluster correlation coefficient of 1% and an assumed coefficient of variation in cluster sizes of 20% (based on average coefficients of variation in actual cluster sizes according to 2016 data).

### Participant recruitment

The study population will consist of all patients with newly registered or previously treated pulmonary TB at eligible and consenting TB units between May 2019 and November 2020 with DS and DR pulmonary TB. All of the eligible patients in the TB units who offer informed consent to participate in the study will be enrolled. A screening log will be kept to identify patients who do and who do not meet the inclusion criteria. A screening tool (Additional file 3) will be used for the assessment of the eligibility and the reasons for non-eligibility will be notified. TB doctors will be responsible for obtaining informed consent from patients to participate in the study. Patients will reach the endpoint when they complete the treatment, die or are lost to follow-up. The recruitment will be stopped by the study coordinators 18 months after the beginning of the trial only—this is to ensure that the minimum sample size is achieved and as much data as possible are collected; the last cohort of DS-TB patients can thus finalize treatment while still within the study period.

### Data collection

We will collect general patient-level data (demographic, socio-economic), outcome data (primary, secondary outcomes) and other TB-related data (diagnosis, referrals) from participating intervention and control facilities. The cohort of patients will be followed and respective data collected in different time periods. See Additional file 4 for the data collection tools, timing, data collection techniques, data sources and responsible persons for data collection.

Various data sources will be used to collect information on the cohort of patients enrolled in the trial:

- Facility-level data—number of TB patients treated by the facility
- Patient-level data:
  - Primary data (individual patient data): TB doctors will be trained in how to conduct patient recruitment and additional ethical procedures. The training will be provided by the researchers for all TB doctors working in the intervention and control sites during the preparatory stage of the study (1 month prior to the trial initiation). Upon enrolment, TB doctors will be responsible for collecting patient socio-demographic data. Data on TB diagnosis, DOT treatment regimen, adherence to treatment and referrals will be collected by a research assistant; the research assistant will be responsible for data extraction through document review (TB registries, patient records). As it is unlikely that facilities will have timely access to data on TB outcomes (treatment success in particular), secondary data will also be consulted
  - Secondary data (cohort data): routine data sources (national TB database from the NCTLD, TB registration journals and forms, and patient records maintained by the TB units) will be used to extract data on the aggregate patient cohorts’ outcomes and other variables including hospitalization of patients and referrals. Secondary data will be compared to primary data to evaluate completeness and accuracy of primary data on adherence; secondary data will be used to estimate DS-TB treatment success. A database manager, an employee of the NCTLD who has access to the national TB database under his routine responsibilities, will be contracted under the study and will be responsible for data extraction from the national TB database.

### Data management and confidentiality

The study results will never present any identified information, all paper-based data with personal identifiers will be stored in a secure separate place and all electronic databases will be encrypted and stored in a password secured computer and on the back-up server. Initial and refreshment trainings will be provided to key study staff on data confidentiality and security. Data security will be monitored by the project manager on a regular basis.

All study data will be stored at Curatio International Foundation (CIF). All electronic and paper-based data will be kept securely, password-protected and locked, with limited access only for authorized persons. All data will be kept on secure drives and backed up daily to minimize loss of data in the long term. All study materials including project administrative documents will be stored and then archived in a locked place.

A metafile for quantitative databases will be created and stored in the same file as the databases. The metafile will include information such as sample ID, instrument settings, list of variables and operator ID. Only authorized persons will have access to these metafiles.

In accordance with the CIF data management policy, all data will be stored at the CIF office in a secure place for an additional 5 years after the project closure. Paper documents will be destroyed through shredding. Electronic data will be de-identified and securely stored on the CIF server.

### Auditing

A Data Monitoring Committee (DMC) will be established. The DMC is independent of the study organizers. Members of the DMC will be representatives from the NCTLD, NCDC and Health Research Union (HRU). The DMC will meet at least once a year, or more often as appropriate. During the period of recruitment to the study, interim analyses will be supplied, in strict confidence, to the DMC, together with any other analyses that the committee may request. The research funder (MRC-UK) may audit the trial in order to reassure itself of the reliability of the data gathered and the ethical conduct. Audit(s) will be carried out by individual(s) entirely independent of the trial and the MRC will define the level of audit required.

### Data analysis

A detailed analysis plan will be developed prior to completion of data collection and locking of the trial database. The analysis plan will include details on the populations to be included in each analysis, general principles for analysis (on level of confidence, *p* values, adjustment for multiple testing, handling of missing data), the analytic approach (anticipated to be intention to treat) as well as analyses for primary and secondary outcomes (adjusted, unadjusted and wider sensitivity and subgroup analyses).

Briefly, we anticipate conducting analyses based on intention-to-treat principles using all complete case data available. Analyses will be conducted at both facility and individual participant levels; for the latter we will allow for clustering of individuals into facilities. Unadjusted analyses will examine differences in primary and secondary outcomes among study groups. However, we will examine baseline imbalance across study groups, and in adjusted analyses account for these in addition to facility weighting (by inverse variance), study strata and/or covariates used in the randomization procedures.

We will examine data missingness; if the missing data rate is more than 5%, we will attempt multiple imputation. Subgroup analyses will be conducted for primary and secondary outcomes, and will consider the facility location (urban vs. semi-urban), the private (including providers with multiple facilities vs. single) or public health care provider and the patient comorbidity subgroup (e.g. patients with HIV or diabetes, among others).

### Alternative analyses

While randomized control trials (RCTs) are the gold standard for evaluating intervention effectiveness, we acknowledge that effectiveness estimates sourced from trial analyses may not reflect the full impact of the intervention on the TB population in Georgia; that is, despite adopting a pragmatic design, the trial is restricted to evaluating effectiveness among incident cases only and excludes patients presenting with the most severe forms of tuberculosis (MDR-TB or those hospitalized for substantive periods). Effectiveness estimates gleaned may thus not apply to the entire patient population affected by the intervention. To address this, a second impact evaluation study is planned using a controlled interrupted time-series design. The study will focus on the same outcomes as for the trial, but adopt a wider perspective and consider outcomes for all patients with pulmonary TB treated in the units of interest. Similar to the trial, data will be sourced from the TB reporting system; we will include quarterly outcome estimates from the period 2017–2020 and the first quarter of 2021 (17 data points), and explore the impact of the intervention on outcomes of interest via segmented regressions. We hypothesize that the intervention would lead to a lagged change in TB outcomes and likely affect both the trend level and gradient over time.

A pre-test of the intervention has been conducted in two TB units between January and April 2019. The purpose of the pre-test was to test instruments/tools and to field-test the intervention. This will also to help fine-tune monitoring processes. Patients with TB satisfying the inclusion criteria were enrolled in the pre-test and followed up for 3 months. Patients recruited during this period will not contribute to the trial outcomes. The intervention will continue in the pre-test TB units without interruption.

### Time schedule

Please see Additional file 4.

## Discussion

### Committees

A steering committee will oversee trial management and implementation; a Data Management Committee will oversee all issues relating to data collection, quality and monitoring. Details of these committees are available in Additional file 5.

### Protocol review

The current protocol has been reviewed by the steering committee of the trial as well as trial funders and the ethics boards of the institutions involved.

### Harms

There are no harms anticipated for either patients or providers from taking part in the study; post-trial care procedures are not relevant. It is not anticipated that interviewing patients about their socio-economic status will cause any distress except for minimal emotional discomfort. No financial or other types of incentives will be provided as a reward for the study participation (beyond the intervention support itself).

### Protocol amendments

Any modifications to the protocol which may impact on the conduct of the study or potential benefit of the patient or may affect patient safety, including changes of study objectives, study design, patient population, sample sizes, study procedures or significant administrative aspects, will require a formal amendment to the protocol. Such an amendment will be agreed upon by CIF and Queen Margaret University (QMU) and approved by the Bioethics Committee at the National Centre for Disease Control of Georgia and the QMU institutional review boards, prior to implementation, and notified to the health authorities in accordance with local regulations. Administrative changes of the protocol are minor corrections and/or clarifications that have no effect on the way the study is to be conducted. These administrative changes will be agreed upon by CIF and QMU and will be documented in a memorandum. The Bioethics Committee at the National Centre for Disease Control of Georgia will be notified of administrative changes at the discretion of CIF.

### Dissemination of trial results

Trial investigators will communicate results to international and national academic and policy audiences internationally via conferences and preparation of published papers, to national-level policy-makers in Georgia via policy briefs and stakeholder meetings, and to lay audiences via lay summary materials (e.g. posters, videos) to be published on the trial website. The scientific integrity of the study requires that results of the trial are adequately shared. Primary outcome and other study papers, abstracts and presentation will be developed based on the trial results. Each product must be shared with and approved by the Coordinating Center before dissemination.

There are no restrictions on preparation of publications arising from this trial. All study investigators will be eligible for authorship, depending on contributions to the trial, its analysis and manuscript preparation. The study team will have exclusive use of the data for 3 years after the project ends. This embargo period is requested to allow time for additional analyses and further publication of research findings.

## Trial status

- Protocol date and version: 16 May 2019, version 2
- Recruitment start: 24 May 2019
- Recruitment end: 24 November 2020
- WHO Trials dataset items: see Additional file 6

### Additional files

Additional file 1: Description of intervention models. (DOCX 175 kb)

Additional file 2: SPIRIT 2013 Checklist: Recommended items to address in a clinical trial protocol and related documents (DOC 120 kb)

Additional file 3: Ethics forms and patient screening tool. (DOCX 43 kb)

Additional file 4: Project timeline, data collection matrix and tools. (DOCX 33 kb)

Additional file 5: Steering committee and data management team. (DOCX 14 kb)

Additional file 6: Trial registration in World Health Organization Trial Dataset. (DOCX 25 kb)

## Data Availability

Data sharing is not applicable to this article as it is a protocol for a future trial. The final trial dataset will be accessible to the MoLHSA, Georgia and investigators at CIF and QMU. Findings of analyses conducted will be made available to the wider research project group, including researchers at LSHTM and ITM. The data will be suitable for sharing with some restrictions. The metafile describing datasets will be shared on our website along with a project description. The data manager of the study will have permission to make decisions on data sharing with new users. This will depend on what type of data needs to be shared CIF will ensure that all new users sign the Data Sharing Agreement in which the specific purpose of data usage will be stated.

## Appendix 1

**Table 1:** Facilities eligible to take part in the trial

ID	Region	Urban/semi-urban	Facility operation type	Facility ownership	Facility name
9	Guria	Urban	Integrated	Private	Ozurgeti
14	Imereti	Urban	Specialized	Private	Kutaisi
16	Imereti	Semi-urban	Integrated	Private	Samtredia
29	Kakheti	Urban	Integrated	Private	Telavi
32	Kvemo Kartli	Semi-urban	Integrated	Private	Gardabani
33	Kvemo Kartli	Semi-urban	Integrated	Private	Marneuli
34	Kvemo Kartli	Urban	Specialized	Private	Rustavi
38	Mtskheta Mtianeti	Semi-urban	Integrated	Private	Dusheti
47	Samegrelo-Zemo Svaneti	Semi-urban	Integrated	Private	Khobi
51	Samegrelo-Zemo Svaneti	Semi-urban	Integrated	Public	Senaki
52	Samegrelo-Zemo Svaneti	Semi-urban	Integrated	Private	Tsalenjikha
53	Samegrelo-Zemo Svaneti	Urban	Specialized	Private	Zugdidi
61	Shida Kartli	Urban	Integrated	Private	Gori
65	Tbilisi	Urban	Specialized	Public	NCTLD
66	Tbilisi	Urban	Specialized	Public	TB Unit—# II
67 and 68	Tbilisi	Urban	Specialized	Public	Merged TB Units # III and # V

## Appendix 2

**Table 2:** Georgian tuberculosis treatment guidelines: TB treatment duration by phase and type of patient in Georgia

Type of patient	Treatment duration (months)	Intensive phase duration (months)	Continuation phase duration (months)	Hospitalization duration (months)
DS-TB				
DS-PTB	6	2	4	1.5–2*
DS-extra PTB (TB meningitis)	12	2	10	
DS-extra PTB (skeletal TB)	9	2	7	
DR-TB				
MDR-TB/MDR-RR-TB individualized regimen	20–24	> 8	> 12	4–6
MDR-TB/MDR-RR-TB shorter regimen	9–12	4–6	5	

*It is expected that hospitalization duration will be shortened to 1 month for patients with DS-TB.

*DR-TB* drug-resistant tuberculosis, *DS-TB* drug-sensitive tuberculosis, *MDR-RR-TB* multidrug-resistant rifampicin-resistant tuberculosis, *MDR-TB* multidrug-resistant tuberculosis, *PTB* pulmonary tuberculosis, *TB* tuberculosis

Adapted from [26].

## Abbreviations

CIF: Curatio International Foundation
DMC: Data Monitoring Committee
DOT: Direct observation of treatment
DR-TB: Drug-resistant tuberculosis
DS-TB: Drug-sensitive tuberculosis
GF: The Global Fund
HIV: Human immunodeficiency virus
HRU: Health Research Union
LFU: Loss to follow-up
LMIC: Low- and middle-income country
MDR-RR-TB: Multidrug-resistant rifampicin-resistant tuberculosis
MDR-TB: Multidrug-resistant tuberculosis
MDT: Multidisciplinary team
MoLHSA: Ministry of Labour, Health and Social Affairs
NCDC: National Center for Disease Control and Public Health
NCTLD: National Center for Tuberculosis and Lung Diseases
PBF: Performance-based financing
PHC: Primary health care
PTB: Pulmonary tuberculosis
QMU: Queen Margaret University
Results4TB: Designing and evaluating provider results-based financing for tuberculosis care in Georgia: understanding costs, mechanisms of effect and impact
RIF: Tuberculosis and resistance to rifampicin
TB: Tuberculosis
VOT: Video observed therapy
WHO: World Health Organization
Xpert MTB: Automated diagnostic test to detect presence of mycobacterium
