# Supplementary material for: Addressing challenges in tuberculosis adherence via performance-based payments for integrated case management: protocol for a cluster randomized controlled trial in Georgia
Source: Trials. 2019 Aug 28;20:536. doi: 10.1186/s13063-019-3621-z (PMC6714082; doi:10.1186/s13063-019-3621-z)
Supplement: Supplementary file 1 — Description of intervention models. (DOCX 175 kb) [file 13063_2019_3621_MOESM1_ESM.docx]

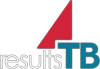
 **Results4TB Project**

**Integrated Care and Results-Based Financing intervention to improve adherence among drug-susceptible and drug-resistant patients with TB in Georgia**

**Description of the Intervention Package**

# Rationale

1. Lack of horizontal coordination between Primary Health Care (PHC) and TB services at the primary level of healthcare organization.
2. Lack of a cooperation-based multidisciplinary approach to TB management at the primary healthcare level (e.g. engaging family doctors and local health professionals in identifying and managing side effects of TB drugs, etc.)
3. Little financial motivation of TB doctors and nurses, and of the managers of the healthcare organisations
4. Point 1-3 lead to less than ideal adherence to treatment and less than optimal treatment outcomes
5. Threat to sustainability of TB services at the PHC level due to the low interest of private providers in retaining TB services
6. The financial burden of managing side effects caused by TB drugs for TB patients is one of the reasons for treatment interruption. Currently, the National TB Program through its voucher financing scheme covers cost of medicines to manage side effects of TB drugs in all DR-TB patients and cost of diagnostic investigations and specialist consultations in patients receiving new drugs and in other DR-TB patients (pursuant to the guideline). However, diagnostic investigations and consultations to detect and monitor side effects of TB drugs are currently available only in the National Center for Tuberculosis and Lung Disease (NCTLD) in Tbilisi and in selected regional health facilities. Health facilities in other locations fail to provide a full range of services and patients have to seek these services in other health facilities at financial cost.
7. Discussions at the policy level to integrate TB services into PHC.

# Current description of services:

At outpatient level, TB services are provided at TB units by TB doctors and TB nurses. There are currently 68 TB units in the country: 58 semi-urban (located in district centres) and 10 urban TB units. Abkhazia and South Ossetia are out of control territories and therefore the state health programmes are not provided to the population residing in these territories.

Semi-urban units were recently integrated into district and regional level health centres (which also offer general outpatient services), most of which are private (hereafter referred as *integrated*).

Only a few TB units remain as separate public institutions mainly in the capital or are merged with hospitals that do not provide outpatient services (in three main cities of the country). Overall there are 10 such urban centres (hereafter referred as *specialised TB units*). The latter units serve a greater number of patients in total compared to accumulated number of patients receiving services in semi-urban integrated TB units. TB services provided in either type of TB Units does not differ from each other, with exception of a TB unit located at Tbilisi National Centre for Tuberculosis and Lung Diseases (NCTLD) where diagnostic and specialists capacity is higher.

TB units provide services to all TB patients residing in the district center / city and nearby villages. In rural areas DOT is provided by a general primary care nurse under the supervision of the TB doctor from respective TB unit.

General outpatient services, hereafter referred as Primary Health Care (PHC) services^[[1]](#footnote-1)^ are delivered by a family doctor under the state funded Universal Health Coverage (UHC) program (private insurance schemes are less prevalent at the district level). The rural population gets basic PHC services from rural family doctors and general primary care nurse, specialized medical services are offered from district health centers (under the UHC).

Specialised secondary services for TB management are offered via the inpatient department within the National Centre for Tuberculosis and Lung Diseases (NCTLD) in Tbilisi and four cities (Kutaisi, Batumi, Zugdidi and Abastumani).

# Intervention

The intervention is defined as a package of interventions, including Results Based Financing (RBF) of healthcare providers, aimed to improve TB treatment adherence and outcomes in TB patients.

Two models of intervention packages have been proposed considering the current set-up of TB services at the outpatient level:

1. Intervention package for TB units administratively integrated into PHC (semi-urban areas)
2. Intervention package in TB units administratively NOT integrated into PHC - so-called specialized TB services (urban areas)

**Target population**: Drug Sensitive (DS) and Drug Resistant (DR) TB patients

**Level of care:** TB outpatient care

**Primary outcome of the intervention:**

- Improved adherence to TB treatment among DS-TB
- Reduced loss to follow-up among DS-TB
- Improved adherence to TB treatment among DR-TB patients
- Reduced loss to follow-up among DR-TB patients
- Improved treatment success rate among DS-TB patients

**Secondary outcomes of the intervention:**

- Improved treatment success rate among DR-TB patients
- Improved horizontal coordination between PHC and specialized TB services (communication, patient information flow, supervision, shared responsibility on a case management, CME, quality assurance)
- Reduced patient costs associated with the management of side effects caused by TB treatment and of comorbidities
- Improved outcomes of managing comorbidities measured by hospitalization rates due to severe comorbid conditions (diabetes and cerebrovascular conditions).

**Spill-over effect of the intervention**

- Improved detection of TB cases at PHC level due to family doctors improved knowledge on TB and financially motivation to identify and manage TB patients

The table below demonstrates functions of personnel involved in TB care under the current and proposed interventions. Bolded are new functions.

Table 1. Distribution of function under current and proposed interventions

|  | Current service provision | Intervention for TB unit integrated into PHC | Intervention for specialized TB unit |
| --- | --- | --- | --- |
| TB doctor | Case detection  Diagnostics  Case management  Treatment monitoring  Screening for side-effects  Monitoring of side-effects  Management of side-effects (all)  Patient education | Case detection  Diagnostics  Case management  Treatment monitoring  Screening for side-effects  Monitoring of side-effects  Management of side-effects (all)  Patient education  **Counseling on tobacco, alcohol**  **PCC approach**  **Multidisciplinary**  **team work** | Case detection  Diagnostics  Case management  Treatment monitoring  Screening for side-effects  Monitoring of side-effects  Management of side-effects (all)  Patient education  **Counseling on tobacco, alcohol**  **PCC approach**  **Team work** |
| TB Nurse | DOT administration | DOT administration  **PCC approach**  **Multidisciplinary**  **team work** | DOT administration  **PCC approach**  **Team work** |
| Family doctor | Case detection | Case detection  **Case management**  **Screening for side-effects**  **Management of side-effects (mild)**  **Patient education**  **Counseling on tobacco, alcohol**  **PCC approach**  **Multidisciplinary**  **team work** |  |
| Rural Family doctor | Case detection | Case detection  **Screening for side-effects**  **Management of side-effects (mild)**  **Patient education**  **Family members education**  **Counseling on tobacco, alcohol**  **Contacts investigation**  **PCC approach**  **Multidisciplinary**  **team work** | Case detection  **Screening for side-effects**  **Management of side-effects (mild)**  **Patient education**  **Family members education**  **Counseling on tobacco, alcohol**  **Contacts investigation**  **PCC approach**  **Multidisciplinary**  **team work** |
| Rural nurse | DOT administration | DOT administration  **Patient education**  **PCC approach**  **Multidisciplinary**  **team work** | DOT administration  **Patient education**  **PCC approach**  **Multidisciplinary**  **team work** |

# (1) Intervention integrated into PHC

## Facility Type

A PHC facility or a Health Center with an outpatient ward in a district or regional center with an administratively integrated TB care unit and

- with a capacity to ensure access to other specialized services (cardiology, endocrinology, gastroenterology, dermatology, mental health, neurology, otolaryngology, ophthalmology)
- with a capacity to ensure access to diagnostic investigations (biochemistry investigations, ECG, audiometry)

## Description of the Intervention

The intervention will take a Patient Centered Care^[[2]](#footnote-2)^ (PCC) approach with which TB patients will receive integrated, multidisciplinary treatment at the PHC level.

The hypothesis under intervention include:

(1) the creation of a better relationship between TB patient and providers through adopting a Patient Centred Care approach, which should result in engaging the patient in the decision-making process and in better trust, better management of co-morbidities and side effects to TB drugs, which in turns contributes to better adherence to treatment.

***preconditions:***

1. The team is integrated (i.e. well coordinated so that there are minimal or no gaps in service delivery).
2. Other barriers to access are absent or minimal: geographical barrier, psychological barriers, hidden costs to care, etc.

(2) the incentives are supposed to stimulate the TB team to accomplish / improve their duties

***preconditions:***

1. good competences (see training component)
2. working conditions conducive to quality care (incl. having the time, infrastructure to do all the meetings and coordination).

The intervention includes:

- defining new roles and responsibilities of health care providers involved in TB case management (incl., a TB doctor, a family doctor, a DOT nurse, a rural nurse) within the scope of their professional competencies to ensure integrated patient centred approach
- introducing new tools such as
  - facility managers guidelines on implementing the intervention
  - case management plan for patient
  - instruments for monitoring of the integrated team performance
  - instruments for verification of the incentive scheme
- trainings
  - on principles of patient-centred care for all members of a TB team
  - on principles of integrated care, for all members of a TB team
  - on managing side effects of TB treatment, for family doctors and TB doctors
- paying bonuses (incentive payments) to a team based on performance indicator – TB patient retained on the TB treatment )

## Integrated Team

### Composition of an Integrated Team

- TB doctor / pulmonologist (TB case manager)
- DOT nurse (in district centers / cities) or rural nurse
- Family doctor (in district centers / cities) or a rural family doctor

In case a TB doctor is no longer available at the district level (retirement or other reason) there should be a substitute TB doctor working in another district visiting the facility a set number of times a week/month to perform regular duties.

Alternatively, a family doctor can be retrained in providing TB services (with a short-term retraining course).

### Goal of work of an Integrated Team:

To ensure TB treatment adherence at the outpatient level by providing multidisciplinary, patient-centered care to a TB patient.

### Principles of Work of an Integrated Team:

- patient-centered care approach
- confidentiality
- teamwork to attain goals

## Table 2 Roles of health professionals affiliated to an integrated team and an incentive scheme

| Roles and responsibilities in detecting, diagnosing and managing TB cases | Participates in an incentive scheme | | | | | |  |  |
| --- | --- | --- | --- | --- | --- | --- | --- | --- |
|  | TB doctor / pulmonologist | DOT nurse in district center | Family doctor in district center / city | Rural family doctor | Rural nurse | Manager | Epidemiologist | Coordinator |
| **Clinical** |  |  |  |  |  |  |  |  |
| Identification of TB suspect cases (passive detection) | √ |  | √ | √ |  |  |  |  |
| Active detection of TB suspect cases in high-risk groups for TB (HIV patients, pulmonary TB patients contacts; a guideline to define a strategy for TB case detection in other risk groups is under development) | √ |  | √ | √ |  |  |  |  |
| Antibiotic therapy to differentiate among specific and non-specific processes | √ |  | √ | √ |  |  |  |  |
| Referral of TB suspect cases to a TB doctor |  |  | √ | √ |  |  |  |  |
| Diagnostic tests on TB, HIV and Hepatitis C | √ |  |  |  |  |  |  |  |
| Prescribing TB treatment | √ |  |  |  |  |  |  |  |
| Initial TB counseling during a joint appointment with a patient | √* (lead) | √ * | √ * | √ * | √ * |  |  |  |
| Patient counseling during treatment – TB | √ | √ |  |  | √ |  |  |  |
| Patient counseling during treatment – tobacco, alcohol, harmful habits | √* |  | √ * | √ * |  |  |  |  |
| Administering treatment - DOT |  | √ |  |  | √ |  |  |  |
| Monitoring of treatment (assessing treatment outcomes) | √ |  |  |  |  |  |  |  |
| Screening for side effects (patient interview) | √ | √ | √ | √ | √ |  |  |  |
| Monitoring of side effects (in DR-TB cases in line with annex #7 of the protocol, in DS-TB cases - according to the TB treatment guideline) | √ |  |  |  |  |  |  |  |
| Management of side effects: |  |  |  |  |  |  |  |  |
| Mild side effects (e.g. initiation of a symptomatic treatment) | √ |  | √ | √ |  |  |  |  |
| Mild-to-severe side effects | √ |  |  |  |  |  |  |  |
| Referral to other health specialist (any, except for TB doctor) according to the protocol | √ |  |  |  |  |  |  |  |
| Management of comorbidities |  |  | √ | √ |  |  |  |  |
| In DR-TB cases follow-up after treatment completion every 6 months for next 24 months | √ |  |  |  |  |  |  |  |
| Counseling and education of household members in district centers / cities |  |  | √ * |  |  |  | √ |  |
| Counseling and education of household members in rural areas |  |  |  | √ * | √ * (education) |  |  |  |
| Epidemiological investigation of contacts of newly detected TB cases  (communication with an epidemiologist) though contacts visiting TB unit | √ |  |  |  |  |  |  |  |
| Epidemiological investigation of contacts of newly detected TB cases in rural areas through home visits (communication with an epidemiologist) |  |  |  | √ * (lead) |  |  |  |  |
| Epidemiological investigation of contacts of newly detected TB cases in district centers / cities through home visits |  |  |  |  |  |  | √ (lead) |  |
| **Organizational** |  |  |  |  |  |  |  |  |
| Coordination of activities within an integrated team | √ * |  |  |  |  |  |  |  |
| Elaboration of a TB case management plan | √ * |  |  |  |  |  |  |  |
| Coordination of a TB case management plan with management plans of comorbidities | √ |  | √ * | √ * |  |  |  |  |
| Monitoring the implementation of an intervention (attending integrated team meetings) |  |  |  |  |  |  |  | √* |
| Monitoring of DOT completion with checklists, patient surveys |  |  |  |  |  |  |  | √ |
| **Administrative** |  |  |  |  |  |  |  |  |
| Establishment of an integrated team |  |  |  |  |  | **√ *** |  |  |
| Contracting health professionals participating in an incentive payment scheme |  |  |  |  |  | **√ *** |  |  |
| Contracting to acquire needed diagnostic and specialists’ services |  |  |  |  |  | **√** |  |  |
| Monitoring and supervision of an integrated team |  |  |  |  |  | **√ *** |  |  |
| Reporting on performance of intervention to the Social Service Agency |  |  |  |  |  | **√ *** |  |  |

* new function under the intervention package

### Rules of Operation of an Integrated Team

1. A TB doctor coordinates an integrated team with the support of a facility manager
2. An integrated team meets jointly a patient upon the registration of a new TB case. The meeting aims at:
3. developing a TB case management plan in cooperation with the patient
4. giving explanations to the patient and his or her family members about the disease and specifics of its treatment
5. familiarizing a patient with roles and responsibilities of the team members and explaining to him or her whom to address in case of a specific need
6. assessing patient’s attitude towards the treatment and expected barriers and bring in an adherence service if needed
7. giving explanations to the patient and his or her family members about investigation of contacts
8. planning a follow-up meeting
9. Initially, family doctor should be visited once a month along with visiting a TB doctor; later visits shall be carried out as needed. Visits to a family doctor aim at examining chronic comorbidities if any, screening for side effects of TB drugs, counselling on smoking and harmful habits, and planning visits to other specialists.
10. The integrated team convenes once a month to review each TB case. Meetings aim at assessing treatment progress, identifying barriers to treatment adherence and elaborating on joint action strategies (engagement of an adherence service, other specialists, etc.)
11. Referrals to specialists to manage side effects of TB drugs are made by a TB doctor. In case family physician and rural family doctor identify need of specialists, they refer the patient to a TB doctor who refers the patient to specialists. Specialist consultations are financed through the National TB Program.
12. If a rural physician is affiliated to an integrated team he or she has to participate in an initial joint meeting with a TB patient along with a rural nurse. A follow-up coordination can be made through phone communication.

# (2) Intervention Package Tailored to Specialized TB services

This package of interventions for TB will be dedicated solely to standalone TB units which are not linked administratively to a PHC services.

In total there are 9 such TB units: Tbilisi (4 TB units), Rustavi, Kutaisi, Batumi, Zugdidi, and Poti.

## Challenges of Participation of Family doctors in the Specialized TB Services

Although the engagement of family doctors in the intervention at specialized TB services like Tbilisi, Rustavi, Kutaisi, Batumi, Zugdidi, Poti facilities will encourage an integrated approach, it will be associated with the following barriers:

- Additional barrier to a patient to visit a family doctor at a PHC facility located in other geographic area.
- Operational difficulties:
  - Communication of a TB doctor with family doctors. In such facilities a TB doctor manages on average 20 patients each of which is served by his or her own family doctor. This will pose difficulties to implementing integrated work, e.g. to organizing joint meetings and managing communications.
  - Contracting of and payment of bonuses to family doctors by the management.
  - Difficulties in monitoring
- Low bonuses to family doctors due to a low number of patients (on average, one patient) will not build up motivation

Therefore, family doctors CAN NOT be involved in the intervention under this model.

## Package of Intervention

- Introduction of new tools such as
  - facility managers’ guideline on implementing the intervention
  - a case management plan for patient
  - instruments for monitoring, of the team performance
  - instruments for verification of the incentive scheme
- Trainings
  - on principles of Patient Centred Care for TB team
  - on managing side effects of TB treatment for TB doctors and specialists
- paying bonuses (incentive payments) to a team based on performance indicator – TB patient retained on the TB treatment )

## Team

### Composition of a Team

- TB doctor / pulmonologist (TB case manager)
- DOT nurse

Roles and responsibilities of a TB doctor and a DOT nurse are the same as listed above. (see in Table 2)

### Goal of work of a Team:

To ensure TB treatment adherence at the outpatient level by providing, patient-centered care to a TB patient.

### Principles of Work of a Team:

- patient-centered care approach
- confidentiality
- teamwork to attain goals

# Supervision and monitoring of the intervention

*Performed by:* a coordinator and a monitor (from the TB Program and supported by the project).

*Timing and Frequency:* Supervision once per month each TB unit during the first three months of the intervention, subsequent visits will be done for monitoring purposes on a quarterly basis (see supervision and monitoring section).

*Instruments and methods*: See Annex 1 for instruments

- Monitoring of registration forms (under the routine functions)
- Observation of practice using relevant observation instrument
- Patient interview using the “Patient Questionnaire”

# Performance payments

## Structure of performance based payments

Performance based payments should be well balanced among health care personnel to build up motivation, ensure fairness and avoid negative consequences.

The volume of a financial incentive is determined based on the following:

- The number of pulmonary DS-TB and DR-TB patients on outpatient treatment in a facility
- The monthly income of a facility generated through voucher financing (average of vouchers of DS-TB and DR-TB patients) topped up by about 40%
- All players (facility, manager, TB doctor, DOT nurse / rural nurse, family doctor / rural nurse) contribution
- Similar bonus payments in large cities and district centers
- Higher bonus payment for a rural doctor compared to a family doctor in urban area (considering low number of patients in rural areas)
- Higher bonus payment for a facility and a facility manager in an integrated TB unit compared to a specialised TB unit (considering higher volume of work and lower number of patients)
- The facility share intended to cover small additional costs to implement the intervention (e.g. meetings, communication, travel costs etc.).

Performance based payments will be based on the work, measured by an indicator – pulmonary DS-TB and DR-TB patients adherence rate to treatment, fulfilled per month with payments on a quarterly basis.

The amount of the bonus shall be distributed among the personnel, facility and manager in line with the rates indicated in the table (see Table 3).

## Table 3 Performance-based bonuses and rates*

|  |  | Bonus per patient per month (GEL, gross) | | | | | | |
| --- | --- | --- | --- | --- | --- | --- | --- | --- |
| Facility type | Patient | Bonus Amount | Facility | Manager | TB doctor | Family doctor | DOT nurse / rural nurse | Rural Family Doctor |
| Integrated | Urban | 60.84 | 7.58 | 6.74 | 17.36 | 14.41 | 14.74 |  |
|  | Rural | 68.27 | 7.29 | 6.48 | 16.70 |  | 14.18 | 21.84 |
| Specialized | Urban | 37.30 | 3.12 | 2.08 | 17.36 |  | 14.74 |  |
|  | Rural | 56.87 | 3.12 | 2.08 | 16.70 |  | 14.74 | 21.84 |

* The rates were adjusted twice: 1) in December 2018, to account for local currency inflation^[[3]](#footnote-3)^ and decreasing number of TB cases. TB doctors, family doctors, rural doctor rate has increased by 40%, nurses by 50%, integrated facility managers – by 20%, facility rate and facility manger’s rate in specialised facility has decreased considering the workload; 2) In January 2019, after introduction of a Pension reform. This scheme consists of the activation of a so-called “funded pension”, to which the employee, the employer and the state treasury has to make a contribution of 2% of monthly income.

## Indicator used as a basis for performance payments

## Table 4 Indicator passport

| Indicator | Pulmonary DS-TB and DR-TB patients adherence rate to treatment |
| --- | --- |
| Description | The share of pulmonary DS-TB and DR-TB patients adhered to TB treatment from the total number of pulmonary DS-TB and DR-TB patients prescribed to undergo (receiving) TB treatment based on a bacteriologically confirmed or clinically diagnosed diagnosis. |
| Definitions | Adherence to treatment for **pulmonary DS-TB patients**   - - - from the start of the outpatient treatment up to the treatment completion, not more than one missing day per month if a patient visits a facility 8-12 –times during a month to fulfill DOT (DOT is performed 2 or 3 days per week and a patient receives drugs for home-taking during next day(s))     - from the start of the outpatient treatment up to the treatment completion, no single missing day per month if a patient visits a facility 4 – times during a month to fulfill DOT (some patients visit a facility only once a week for DOT and receive drugs for home-taking during next days)   Adherence to treatment for **pulmonary DR-TB patients**   - - - from the start of the outpatient treatment, not more than three missing days per month (DOT is performed 26 days per month - 6 days a week except Sunday). |
| Required data | - Pulmonary DS-TB and DR-TB patients DOT daily performance - Total number of pulmonary DS-TB and DR-TB patients per month |
| Data Sources | - Paper forms:   - TB-01 form (for DS-TB DOT daily performance)   - TB-23 form (for DR-TB DOT daily performance)   - TB-03 journal (DS-TB patients total number)   - TB-02 journal (DR-TB patients total number) - E-Health electronic database (to be finalized, will include similar to paper forms information) |
| Calculation method | Numerator  Pulmonary DS-TB and DR-TB adhered to treatment patients per month  Denominator  Pulmonary DS-TB and DR-TB total number of patients per month  Note: If the treatment is terminated for medical reasons, such patient will not be counted in the indicator (denominator) calculation for the respective period. |
| Responsible for data collection | - DOT nurse - Rural nurse |
| Responsible for data reporting | TB doctor |
| Frequency | Registration: daily  Measurement within facility: monthly  Reporting: quarterly |

- Verification of an Indicator is performed by a purchaser (see verification section)

## Administration of Performance Payments

### Public purchaser – Social Service Agency

The public purchaser Social Service Agency (SSA) will contract health facilities under the intervention. Performance payments will be carried out based on the fulfilment of pre-agreed indicator by a concerned facility.

## Table 5 Performance payment scheme

| Indicator fulfilment | Bonus Volume |
| --- | --- |
| > 85% | 100% |
| 71-84% | 50% |
| < 70% | 0% |

Reporting (by an electronic form) and payments are implemented quarterly. (see Annex 2).

### Health Facility

A health facility under the intervention is obliged to support its implementation and to carry out administration, reporting and monitoring duties.

- Provision of services
  - A health facility must ensure the provision of all required specialist consultations and diagnostic investigations to a patient (pursuant to annex #7 of the protocol) on its own or by procuring services from another facility (integrated).
  - Members of an integrated team should be informed about where diagnostic investigations and specialist consultations needed for TB patients take place (integrated).
  - In the specialized care settings TB doctor should get a patient’s consent and then contact the patient’s family doctor to inform him or her about the patient’s condition (specialized).
- Administration must
  - contract integrated team members working in the facility and elsewhere (family doctor and rural nurse) and determine their performance payments in line with the prescribed schedule (integrated).
  - contract personnel working in the facility (TB doctor and DOT nurse) and determine their performance payments in line with the proposed scheme (integrated).
  - ensure the establishment and operation of an integrated team (integrated).
  - If a patient is a beneficiary of the UHC and uses respective services at another health facility he or she shall be offered to use these services at the same facility where he or she gets TB services for the period of TB treatment. If the patient denies the offer he or she should not be counted in a bonus scheme (integrated).
  - If a TB doctor is substituted by another district specialist contracted by the PHC facility, the management of the latter shall include the specialist in the team and pay a corresponding bonus (integrated).
  - provide for monthly communication expenses of TB doctors (20-25 GEL per specialist) from income generated through facility performance payment arrangements (integrated).
- Reporting
  - report to the SSA on the DOT performance indicator and the bacteriological test result shall be carried out through the electronic module (needs to be modified). (integrated and specialized)
- Monitoring
  - Monitoring is carried out by the management to assure the attainment of outcome indicators and to quality improvements
  - Proposed indicators, e.g.: (tbd.)
    - Attainment of outcome indicators (successfully completed treatment)
    - The number of joint meetings of the integrated team in a given quarter as compared to the number of patients enrolled in the outpatient treatment in the same period.

# Verification

- 1. *Aim*: Performance financial inspection
  2. *Performed by*: the purchaser
  3. *Timing*: Starts with the intervention.
  4. *Frequency:* Quarterly before reporting
  5. *Method*: on the site in the frame of financial inspection

# Supervision and Monitoring

1. *Aim, timing and frequency* :
   1. Supervision is performed monthly during the first three months to provide on-site support and correct mistakes
   2. Monitoring is done with aim to assess quality of the service after the first three months on a quarterly basis
2. *Performed by: a coordinator and a monitor (randomly). The regional coordinator will verify district level TB units, the coordinator from the NCTLD will verify regional level TB units.*
3. *Instruments and methods: (*See Annex 1 for instruments)
   - 1. Monitoring of the registration forms (within the routine function) – **performance monitoring** during a quarter using FM form.

Similar form is used for laboratory information verification: Sputum microscopy test is done for all pulmonary TB patients end of the 5^th^ month of treatment. Result from the laboratory is received on the 6^th^ month of treatment. TB doctor/or nurse will enter the lab test result into the electronic reporting form by the end of the reporting month.

- - 1. Observation of the Team meeting using an “Observation of Practice” OP form – **quality check**. At least one team meeting should be observed during the quarter visit.
    2. Patient interview using a “Patient Questionnaire” PQ form for **quality check**. At least three patients should be interviewed who come to the DOT visit / consultation to a TB unit. If a TB unit has a rural patient one rural patient should be interviewed by phone.

Annex 1 Supervision and monitoring forms

**Registration forms monitoring - FM**

**(from coordinators supervision protocol )**

*Implemented by*: Coordinator

*Aim:*  Supervision – First three months from the start of the intervention

Monitoring - the period three month after from the start of the I ntervention

1. Date (day / month / year) _______________________
2. TB unit registration code _______________________
3. Coordinator name _______________________

| 1. DOT for DS patients (from question 12: TB-01 forms completed accurately and completely) | |
| --- | --- |
| - 1. Yes | 2 |
| - 1. Partly | 1 |
| - 1. No | 0 |
| 1. DOT for DR patients: (from question 13: cards completed accurately and completely) | |
| 1. Yes, or No Resistant patients | 2 |
| 1. Partly | 1 |
| 1. No | 0 |
| 1. Number of sputum microscopy positive DS PTB on the fifth month |  |
| 1. Accuracy and completeness of information on fifth month sputum microscopy result between reporting form and lab registration journal (Tb-04) |  |
| 1. Yes |  |
| 1. No |  |
| FM total score |  |
| Assessment system:   - 3 - 4 (good) - 2 (satisfactory) - 0 - 1 (not satisfactory) |  |

**Observation of Practice - OP**

**of the (Integrated) Team Performance**

*Implemented by*: Coordinator

*Aim:*  Supervision – First three months from the start of the intervention

Monitoring - the period three month after from the start of the intervention, quarterly

1. Date (day / month / year) ___________________
2. TB unit registration code ___________________
3. Coordinator name ___________________
4. Patient ID (from TB-03, TB-02) ___________________
5. Meeting No for the patient ___________________
6. Team composition and participation in the meeting:

| **Personnel** | **General composition**  **(mark with “X”)** | **Attended**  **(mark with "X”)** | **Absence reason if not attended** |
| --- | --- | --- | --- |
| TB doctor |  |  |  |
| DOT nurse |  |  |  |
| Family doctor |  |  |  |
| Rural family doctor |  |  |  |
| Rural nurse |  |  |  |
| Patient (not mandatory if not the first visit) |  |  |  |
| Full presence or absence due to excused reasons | | | 2 |
| Not full presence due to unknown reason | | | 1 |
| Not full presence due to unexcused reason | | | 0 |
| reasons:   1. Illness, other excused reason 2. Did not have time (unexcused if meeting planned in advance) 3. Did not know (unexcused) 4. Reason not known 5. Other ________________________ | | |  |
| 1. The meeting was conducted | | | |
| - 1. very well (matches with or exceeds the formulated functions) | | | 2 |
| - 1. satisfactorily (on average, matches with the formulated functions of the team | | | 1 |
| - 1. unsatisfactorily (with serious shortcomings), specify reasons: | | | 0 |
| 1. ***Question to the team members****:* How does the management (the manager) assist the work of your team? *(ask specifically if responses are not given)* | | | |
| - 1. Explaining team members’ rights and obligations | | |  |
| - 1. Supervising | | |  |
| - 1. Solving problems as they arise | | |  |
| - 1. Offering regular assistance | | |  |
| - 1. No assistance | | |  |
| - 1. Other ______________________ | | |  |
| 1. ***Question to team members:*** What problems do you experience in the work of the team? *(do not read out, match answers)* | | | |
| - 1. Difficulties in communication | | |  |
| - 1. Family doctor / rural doctor / rural nurse cannot manage to attend | | |  |
| - 1. No assistance from the health facility | | |  |
| - 1. Problems with conducting diagnostic investigations | | |  |
| - 1. Problems with specialist referrals | | |  |
| - 1. Other _________________________________ | | |  |
| OP Total score | | |  |
| Assessment system   - 3-4 (good) - 2 (satisfactory) - 0-1 (unsatisfactory) | | |  |

**Patient Questionnaire - PQ**

**Tuberculosis outpatients care quality assessment**

*Implemented by*: Coordinator

*Aim:*  Supervision – First three months from the start of the intervention

Verification - the period three month after from the start of the intervention

1. Date (day / month / year) ______________________
2. TB unit registration code ______________________
3. Coordinator name ______________________
4. Patient (underline) urban / rural
5. Interviewed (underline) face to face / by phone

| 1. For how long do you receive TB treatment? For _____ months | |
| --- | --- |
| 1. For how long (months) your treatment is going to last? | |
| 1. Answers correctly | 1 |
| 1. Answers incorrectly or does not know | 0 |
| 1. How many tablets do you take and how many times a day? | |
| - 1. Answers correctly | 1 |
| - 1. Answers incorrectly or does not know | 0 |
| 1. How many type of medicines do you take? Which medications are you taking? | |
| - 1. Both answeres correct | 2 |
| - 1. Partly correct (e.g. could not specify medications) | 1 |
| - 1. Boths answeres incorrect ot does not know | 0 |
| 1. How frequently do you undertake sputum testing? | |
| - 1. Answers correctly | 1 |
| - 1. Answers incorrectly or does not know | 0 |
| 1. Please, tell me how TB is transmitted? | |
| - 1. Answers correctly | 1 |
| - 1. Answers incorrectly or does not know | 0 |
| 1. Please, tell me when and how to use a mask? | |
| - 1. Answers correctly | 1 |
| - 1. Answers incorrectly or does not know | 0 |
| 1. Do you have TB treatment plan? Please, show it to me: | |
| - 1. Has | 2 |
| - 1. Do not have it at the moment (lost, etc.) | 1 |
| - 1. Never had | 0 |
| 1. *(If patient has the TB treatment plan)* Is this plan informative /useful to you? | |
| - 1. Yes, why? | |
| - 1. No, why? | |
| 1. Please, list all healthcare professionals who talks and provides advise to you during TB treatment *(do not read out, match answers)* | |
| - 1. TB doctor | 1 |
| - 1. TB nurse or Rural doctor | 1 |
| - 1. Family doctor in an outpatient clinic / center or Rural family doctor | 1 |
| - 1. Specialist | 1 |
| 1. Please, tell me what your complaints are and what care you receive to reduce these complaints. | |
| __________ months / weeks ago (underline) |  |
| 1. Please, tell me what your complaints are and what care you receive to reduce these complaints. | |
| 1. Overall how would you rate the TB care you receive? *(read)* | |
| - 1. Excellent |  |
| - 1. Good |  |
| - 1. Fair |  |
| - 1. Poor |  |
| - 1. Very poor |  |
| 1. Do you have any barriers in continuation of care, and if yes please list (*do not read out, match answers, multiple answers are possible)* | |
| - 1. No barriers |  |
| - 1. Geographical barrier |  |
| - 1. Financial barrier related to drugs for side effect management |  |
| - 1. Financial barrier related to tests |  |
| - 1. Financial barrier related to specialists services |  |
| - 1. Difficulty to synchronise with work |  |
| - 1. Stigma |  |
| - 1. Other (specify) |  |
| PQ Total scores |  |
| Assessment system:   - 9 - 13 (good) - 5 - 8 (satisfactory) - 0 - 4 (unsatisfactory) |  |

**Total Scores**

| 1. FM (registration forms monitoring) score |  |
| --- | --- |
| 1. OP (observation of practice) score |  |
| 1. PQ (patient questionnaire) average scores |  |
| Sum of scores |  |
| Assessment system   - 15 - 21 (good) - 7 -14 (satisfactory) - 0- 6 (unsatisfactory) |  |

**Annex 2. Reporting Form**

**
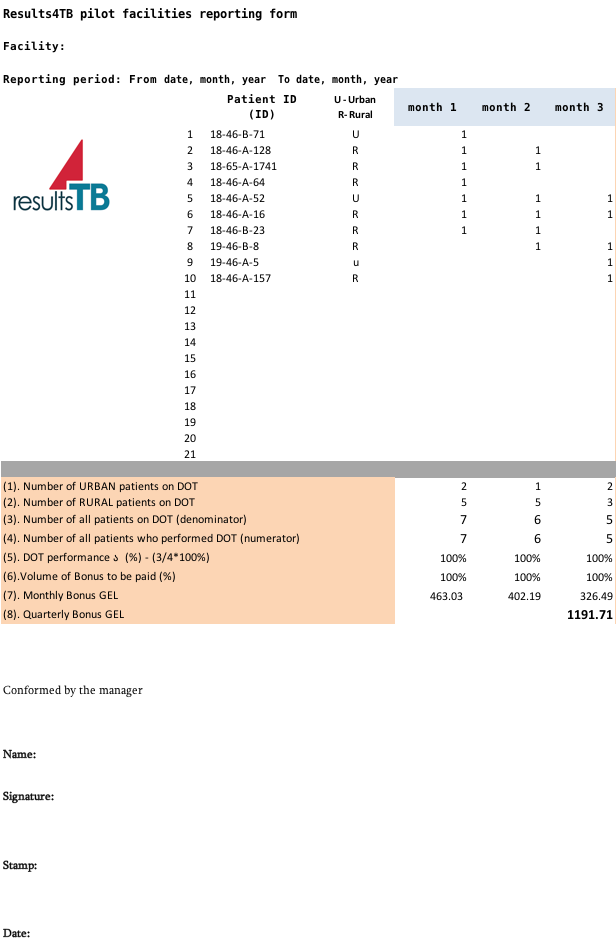
**

1. Even though the services offered do not fully comply with the PHC requirements [↑](#footnote-ref-1)
2. “a holistic model of care delivery that considers the patient as the central figure in the process of continuum of care” (O’Donnell MR, 2016) [↑](#footnote-ref-2)
3. Official inflation rate from July 2017 to November 2018 – 4.7%. source: geostat.ge [↑](#footnote-ref-3)
