# Supplementary material for: Addressing challenges in tuberculosis adherence via performance-based payments for integrated case management: protocol for a cluster randomized controlled trial in Georgia
Source: Trials. 2019 Aug 28;20:536. doi: 10.1186/s13063-019-3621-z (PMC6714082; doi:10.1186/s13063-019-3621-z)
Supplement: Supplementary file 3 — Ethics forms and patient screening tool. (DOCX 43 kb) [file 13063_2019_3621_MOESM3_ESM.docx]

## Information sheet for Health Facility Manager - (Ethics-IF-M)

(Information is given by a researcher and a copy of the form is delivered to the facility manager)

*Study number:* ISRCTN14667607

*CIF Researcher: Ivdity Chikovani, Phone: +995 32 225 31 04, email:* [*i.chikovani@curatio.com*](mailto:i.chikovani@curatio.com)*, address: 3 Kavsadze str. Office #5. Tbilisi, 0179, Georgia;*

*ITM researchers’ names and contact details: Bruno Marchal:* [*bmarchal@itg.be*](mailto:bmarchal@itg.be)*; Ariadna Nebot,* [*anebot@itg.be*](mailto:anebot@itg.be)

*LSHTM Researcher: Anna Vassal:* [*Anna.Vassall@lshtm.ac.uk*](mailto:Anna.Vassall@lshtm.ac.uk)

*QMU Researcher: Predrag Duric:* [*PDuric@qmu.ac.uk*](mailto:PDuric@qmu.ac.uk)

*Local IRB committee- committee -board chairman: Marina Topuridze , Phone: +995 32 239 89 46,  email:* [*irb.ncdc@gmail.com*](mailto:irb.ncdc@gmail.com) *or committee administrator: Tamta Komakhidze, Phone: +995 32 239 89 46, email:* [*tamtakom@gmail.com*](mailto:tamtakom@gmail.com)*, address: 9 M. Asatiani str. Tbilisi, 0177, Georgia.*

[Greetings] My name is _______ , researcher from Georgia based organization Curatio International Foundation. Our organization is conducting a research project “Integrated Care and Results-Based Financing intervention to improve adherence among patients with drug-susceptible and drug-resistant TB in Georgia” in collaboration with the Ministry of Health, Labour and Social Affairs of Georgia and international partners: Queen Margaret University Edinburgh, London School of Hygiene and Tropical Medicine and Antwerp Institute of Tropical Medicine.

With aim to improve TB treatment success among patients with TB the Ministry of Health and Social Affairs of Georgia in collaboration with TB key stakeholders in the country designed an *Integrated Care and Results Based Financing Intervention* that will be piloted in 10 TB service providers in the country. The Intervention will target facility managers, TB specialists and family health care providers to stimulate provision of comprehensive, patient-oriented and integrated care to the patient. Details of the intervention are described in the MoLHSA decree (indicate decree number).

Implementation of the intervention is accompanied with a study to generate evidence on intervention impact on adherence and treatment success rates, costs and cost-effectiveness of the intervention, and how it works in different contexts in Georgia. The study results will inform the design of the provider payment mechanisms under the National TB program and strengthen TB services in the country.

Your healthcare facility has been chosen to participate in this intervention and the study.

There are several components of the study: 1) the Trial component will look at the patients with TB accomplishment of the treatment regimen and their treatment success rate; 2) the Cost-Effectiveness study will evaluate whether the RBF intervention is effective from economic standpoint; 3) the Realist Evaluation component will investigate the factors that influence RBF model implementation from different perspectives.

In the frame of the Trial component we need to recruit patients with TB and follow-up their treatment adherence and treatment outcomes. For this purposes access to their medical records and TB program registration/ reporting forms will be required. For Cost-Effectiveness and Realist Evaluation components we need to collect information from the facility, from managers and health care providers involved in the implementation of the intervention as well as from the patients with TB.

The study results will never identify the facility. The study results may be used for other studies related to TB or economic aspects, or realist evaluation of health services and the data without personal identifiers will be shared publicly.

The study will last for 24 months, and our researchers will be visiting your facility from time to time.

There are no direct benefits for your facility resulting from their participation in this study. The patients treated in your facility may potentially benefit in the sense that the findings may inform to better facility management, policy implementation and quality of care at the facility and lead to measures to improve adherence.

You can choose not to participate. If you decide not to participate in the study there will be no consequences neither for you nor for you institution. If you decide to participate in the study, at any time the facility you represent can withdraw from the intervention and the study without giving a reason and without affecting normal care and management in the facility.

We will seek informed consent individually from all individuals involved in the study.

If you have any questions on this study in the future or would like further information you can contact using the contact details at the top of this sheet. Thank you for your kind support and willingness.

## Enrolment consent form for Health Facility Manager (Ethics-CF-M)

Study number: ISRCTN14667607

Date of consent:

Site:

Facility name:

Manager name representing the facility:

Please initial box if you agree:

I have read/ been read the information sheet concerning the

“Integrated Care and Results-Based Financing intervention to improve adherence among patients with drug-susceptible and drug-resistant TB in Georgia”.

Study and understand what will be required of me if I take part in the intervention and the study.

My questions concerning this study have been answered by:

.....................................................................................................

I understand that participation in the study is voluntary.

I understand that at any time, the facility I represent can withdraw from the study without giving a reason and without affecting normal care and management in the facility.

I understand that there are no direct benefits for the facility resulting from their participation in this study.

I am willing to allow access to the registries, medical notes to ensure that the study is being carried out correctly but understand that strict confidentiality will be maintained.

I agree for the data from this study to be made public understanding that strict confidentiality will be maintained and there is no risk of linking the data to our health care facility.

I agree the facility is enrolled in the study.

**Health facility manager name (print) Health facility manager signature Date**

**Name of study staff conducting Study Staff signature Date**

**consent discussion (print)**

**NB One copy for the facility manager and one for researcher**

## Information sheet for patients for recruitment in the study - (Ethics-IF-Pt)

(Information is given by a doctor and a copy of the form is delivered to the participant)

*Study number:* ISRCTN14667607

*CIF Researcher: Ivdity Chikovani, Phone: +995 32 225 31 04, email:* [*i.chikovani@curatio.com*](mailto:i.chikovani@curatio.com)*, address: 3 Kavsadze str. Office #5. Tbilisi, 0179, Georgia; or*

*ITM researchers’ names and contact details: Bruno Marchal:* [*bmarchal@itg.be*](mailto:bmarchal@itg.be)*; Ariadna Nebot,* [*anebot@itg.be*](mailto:anebot@itg.be)

*LSHTM Researcher: Anna Vassal:* [*Anna.Vassall@lshtm.ac.uk*](mailto:Anna.Vassall@lshtm.ac.uk)

*QMU Researcher: Predrag Duric:* [*PDuric@qmu.ac.uk*](mailto:PDuric@qmu.ac.uk)

*Local IRB committee- committee board chairman: Marina Topuridze , Phone: +995 32 239 89 46,  email:* [*irb.ncdc@gmail.com*](mailto:irb.ncdc@gmail.com) *or committee administrator: Tamta Komakhidze, Phone: +995 32 239 89 46, email:* [*tamtakom@gmail.com*](mailto:tamtakom@gmail.com)*, address: 9 M. Asatiani str. Tbilisi, 0177, Georgia.*

I am doctor ________, supervising you on the TB treatment. Research organization from Georgia Curatio International Foundation is conducting a research project “Integrated Care and Results-Based Financing intervention to improve adherence among patients with drug-susceptible and drug-resistant TB in Georgia in collaboration with the Ministry of Health, Labour and Social Affairs of Georgia and international partners: Queen Margaret University Edinburgh, London School of Hygiene and Tropical Medicine and Antwerp Institute of Tropical Medicine.

The study results will help to develop recommendations for the policy makers how to strengthen TB services in the country.

You have been selected to participate in the study. Participation implies that researchers will have access to your medical records and they might approach you for additional questions on Tb diagnostics and treatment related costs and quality of received care. No additional medical intervention would be required from you.

If you agree to participate in the study your name will not appear anywhere, and the study results will never identify you. Collected data will be stored in a secured place and only researchers will have access to them.

There are no direct benefits for you resulting from their participation in this study. You may potentially benefit in the sense that the findings may inform to better facility management, policy implementation and quality of care at the TB clinic and lead to measures to improve adherence.

Taking part is your choice; you can choose not to answer any of the questions or request to stop at any time without giving explanation.

If you decline to participate, there will be no consequence for you and you will receive whatever care and treatment you need at the health facility currently or in the future. If you refuse you will not lose any benefit that you normally get.

If you have any questions on this study in the future or would like further information you can contact researchers using the contact details at the top of this sheet.

The results may be used for other studies related to TB or economic aspects of use of health services and the data without personal identifiers will be shared publicly.

Thank you for your kind support and willingness.

## Enrolment consent form for patients (Ethics-CF-Pt)

Study number: ISRCTN14667607

Date of consent:

Site:

Participant initials:

Male/ Female:

**Please initial box if you agree:**

I have read/ been read the information sheet concerning the

“Integrated Care and Results-Based Financing intervention to improve adherence among patients with drug-susceptible and drug-resistant TB in Georgia”

study and understand what will be required of me if I take part in the study.

My questions concerning this study have been answered by:

.....................................................................................................

I understand that participation in the study is voluntary.

I understand there are no direct benefits for me resulting from my participation in this study.

I understand that at any time, I may withdraw from this study without

giving a reason and without affecting my normal care and management.

I am willing to allow access to my medical records to ensure that the study is being carried out correctly but understand that strict confidentiality will be maintained.

I agree for the data from this study to be made public understanding that strict confidentiality will be maintained and there is no risk of linking the data to me.

Participant name (print) Participant signature Date

Name of study staff conducting Study Staff signature Date

consent discussion (print)

**NB One copy for participant and one for researcher**

**Patient Eligibility and Enrolment form - (PEE)**

*(To be filled out by TB doctor)*

1. TB unit ID ____________________
2. Intervention type*:* Integrated / Specialised

**Section 1. Eligibility** *Complete section 1 regardless of whether the person is ineligible or declines consent*

1. TB unit name __________________________________
2. TB unit location District center ______________ City ___________________
3. TB doctor name ___________________________________
4. Screening Date _____ / _____ / _____ dd/mm/yy
5. Patient ID (unique code assigned under TB program) _____________________________

**Eligibility status checklist**

*After the first positive answer under column B “Not eligible” consider the patient as not eligible and go to 22*

| Criteria | Eligible (column A) | | | | Not eligible (column B) |
| --- | --- | --- | --- | --- | --- |
| 1. Age _____________ (years) |  | Aged ≥18 |  | Aged <18 | |
| 1. TB diagnosed |  | Bacteriologically confirmed by a direct sputum smear microscopy, culture or Gene Xpert MTB/RIF OR  Clinically diagnosed by an X-ray, histological or morphological changes |  | Neither bacteriologically confirmed nor clinically diagnosed TB case | |
| 1. Type of TB by location |  | Pulmonary |  | Extra-pulmonary | |
| 1. Type of TB: DS-TB MDR/RR-TB |  |  |  |  | |
| 1. Date of start experiencing at least one of TB symptoms: cough, drenching night sweats, unintentional weight loss, fever, chest pain, shortness of breath and tiredness 2. _____ / ____ / ____ dd/mm/yy |  |  |  |  | |
| 1. Date of seeking care for TB symptoms to any type of health provider _____ / ____ / ____ dd/mm/yy |  |  |  |  | |
| 1. Date of TB diagnosis _____ / ____ / ____ dd/mm/yy |  |  |  |  | |
| 1. Date of TB treatment initiation _____ / ____ / ____ dd/mm/yy |  |  |  |  | |
| 1. Hospitalization duration: |  |  |  |  | |
| Patients with DS-TB |  | With less than 2 months of hospitalization |  | With more than 2 months of hospitalization | |
| MDR/RR-TB |  | With less than 6 months of hospitalisation |  | With more than 6 months of hospitalisation | |
| 1. Date of TB outpatient treatment initiation _____ / ____ / ____ dd/mm/yy | | |  |  | |
| 1. Assigned to outpatient treatment 2. (start of the study _____ / ____ / ____ dd/mm/yy) |  | Within 1 month period before or After the beginning of the study |  | 1 month before the beginning of the study | |
| 1. Treatment regimen |  | With standard TB treatment regimen according to national TB Management Guidelines |  | Patients known at the start of treatment to require the treatment longer than it is recommended by the national TB Management Guidelines | |
| 1. Patient involved in other clinical studies related to TB treatment |  | No |  | Yes | |
| 1. Will you be living in this area next 6 months? |  | Yes |  | No | |

**Section 2. Enrolment**

| 1. Eligibility (if positive any answer from 8 to 21 under column B “Not eligible”, mark “No”) |  | Yes |  | No |
| --- | --- | --- | --- | --- |

*If “No” thank the patient and finish data collection.*

*If “Yes” Explain to the patient the purpose of the study (see the Information for patients) and ask the patient to join the study. If the patient agrees, give the written consent form to the patient and write the patient ID on it (same as in q. 7).*

| 1. Did the patient give written consent to participate in the study? |  | Yes (*go to Section 3. Locator)* |  | No |
| --- | --- | --- | --- | --- |
| *If “No”* |  |  |  |  |
| 1. What is the reason for not consenting to participate in the study? 2. Not interested to participate 3. Want to think about (offer to her/him to participate during the next visit if not more than 1 month has passed after treatment initiation) 4. Other (indicate): | | | | |
| *Thank the patient and finish data collection.* | | | | |

*Go to Locator*

**Locator**

**Instruction (*Locator form is stored separately)***

*(To be filled out by researcher)*

1. TB unit ID ____________________
2. Intervention type*:* Integrated / Specialised

*(To be administered by a TB doctor)*

1. Patient ID (unique code assigned under TB program) _____________________________
2. Patient date of birth _____ / ____ / ____ dd/mm/yy
3. Gender
   1. Male
   2. Female
4. Address ___________________________________
5. Cell Phone number ___________________________________
6. Other Phone number ___________________________________

## *Go to Socio-Economic Status (SES) form*
