# Supplementary material for: Addressing challenges in tuberculosis adherence via performance-based payments for integrated case management: protocol for a cluster randomized controlled trial in Georgia
Source: Trials. 2019 Aug 28;20:536. doi: 10.1186/s13063-019-3621-z (PMC6714082; doi:10.1186/s13063-019-3621-z)
Supplement: Supplementary file 4 — Project timeline, data collection matrix and tools. (DOCX 33 kb) [file 13063_2019_3621_MOESM4_ESM.docx]

Trial Gant Chart (Intervention and data collection timing)

| Activity | | Pre-Study | | Study | | | | | | | | | | | | | | | | | | | | | | | | Close-out | |
| --- | --- | --- | --- | --- | --- | --- | --- | --- | --- | --- | --- | --- | --- | --- | --- | --- | --- | --- | --- | --- | --- | --- | --- | --- | --- | --- | --- | --- | --- |
| Year | | 2018 | | | | | 2019 | | | | | | | | | | | | 2020 | | | | | | | | |  |  |
| Time-point | | -t _2-4_ | -t _1-0_ | t_1_ | t_2_ | t_3_ | t_4_ | t_5_ | t_6_ | t_7_ | t_8_ | t _9_ | t_10_ | t_11_ | t_12_ | t_13_ | t_14_ | t_15_ | t_16_ | t_17_ | t_18_ | t_19_ | t_20_ | t_21_ | t_22_ | t_23_ | t_24_ | t_25_ | t_26-30_ |
| Months | | 5-7 | 8-9 | 10 | 11 | 12 | 1 | 2 | 3 | 4 | 5 | 6 | 7 | 8 | 9 | 10 | 11 | 12 | 1 | 2 | 3 | 4 | 5 | 6 | 7 | 8 | 9 |  |  |
| **PRETEST** | | X |  |  |  |  |  |  |  |  |  |  |  |  |  |  |  |  |  |  |  |  |  |  |  |  |  |  |  |
| **PREPARATION** (training on enrolment and ethics) | |  | X |  |  |  |  |  |  |  |  |  |  |  |  |  |  |  |  |  |  |  |  |  |  |  |  |  |  |
| **INTERVENTION** | |  |  |  |  |  |  |  |  |  |  |  |  |  |  |  |  |  |  |  |  |  |  |  |  |  |  |  |  |
| **ENROLMENT** (Informed consent, allocation) Tools: PEE (f) | |  |  | X | X | X | X | X | X | X | X | X | X | X | X | X | X | X | X | X | X |  |  |  |  |  |  |  |  |
| **DATA COLLECTION (C) and ANALYSES (A)** | | | | | | | | | | | | | | | | | | | | | | | | | | | | | |
| TRIAL (Tools) | |  |  |  |  |  |  |  |  |  |  |  |  |  |  |  |  |  |  |  |  |  |  |  |  |  |  |  |  |
| SES (f) | |  |  | C | C | C | C | C | C | C | C | C | C | C | C | C | C | C | C | C | C |  |  |  |  |  |  | A | A |
| PT, FT (e) | |  |  |  |  |  |  |  | C | C |  |  |  |  | C | C | A |  |  |  | C | C | A |  |  |  | C | C | A |
|  | _(f) – collection through face-to-face interview; (e) – data extraction from registries, databases; SES - Socio Economic Status form, PEE – Patient Eligibility and Enrolment form, PT – Patient Trial form, FT – Facility Trial form._ | | | | | | | | | | | | | | | | | | | | | | | | | | | | |

Trial Data collection matrix

| Tools | Data source | Content | Time period | Responsible for data collection | Data collection technique |
| --- | --- | --- | --- | --- | --- |
| PEE, SES | Patient | Patient eligibility screening, (incl. informed consent); socio-economic data | Enrolment | TB doctors | Face to Face interview |
| PT, FT | TB registries, patient records | Outcomes; diagnosis & treatment data | Four times | Research assistant | Data extraction |
| PT | National TB database | Outcome data | Four times | Database manager | Data extraction |
| _SES - Socio Economic Status form, PEE – Patient Eligibility and Enrolment form, PT – Patient Trial form, FT – Facility Trial form._ | | | | | |

**Socio-Economic Status form - (SES)**

*(Administered by a TB doctor upon patient enrolment)*

| 1. TB unit ID *(filled out by researcher)* ____________________ |
| --- |
| 1. Intervention type *(filled out by researcher):* Integrated / Specialised |
| 1. Patient ID (unique code assigned under TB program) _____________________________ |
| 1. What is your highest level of education?    1. None    2. Primary (1- 4 classes)    3. Secondary (school, technical school, vocational school)    4. Incomplete Higher    5. Higher |
| 1. What is your marital status?    1. Married / Living together    2. Divorced/ Separated for ever    3. Widow/ widower    4. Has never been married    5. Other (please indicate) ________ |
| 1. How many people do regularly sleep in your house, including you?   Please indicate ______ |
| 1. Are you currently employed?    1. Student without paid job    2. Have a formal job    3. Informal employment    4. Retired/ disabled    5. Unemployed    6. Housewife |
| 1. How much do you estimate is the average monthly income of your household?    1. Less than 100 GEL    2. 100-299 GEL    3. 300-499 GEL    4. 500-699 GEL    5. 700-999 GEL    6. 1000 GEL and more |
| 1. Are you an internally displaced person or refugee? 2. Yes 3. No 4. No response |
| 1. Do you (your household) receive governmental social assistance for social vulnerability?    1. Yes    2. No    3. Don’t know 2. 99 No response |

**Patient Trial form - (PT)**

*Administered by research assistant and data manger, data collection from the document review, TB database*

| 1. TB unit ID ____________________ | | | |  |
| --- | --- | --- | --- | --- |
| 1. Intervention type*:* Integrated / Specialised | | | |  |
| 1. Patient ID (unique code assigned under TB program) _____________________________ | | | | |
|  | | | Data source | |
| 1. Sputum microscopy (at the time of diagnosis)    1. Smear +    2. Smear -    3. Not performed | | | Pr. TB database  Sc. TB10/12 form | |
| 1. Xpert MTB/RIF test (at the time of diagnosis)    1. MTB       1. MTB(+)       2. MTB(-)       3. Test without result    2. RIF susceptibility       1. RIF resistant       2. RIF susceptible       3. RIF susceptibility not defined    3. Not performed | | | Pr. TB03/02 journals  Sc. TB01 form | |
| 1. Culture (at the time of diagnosis)    1. Positive       1. Resistant (S, H, R, E, Z, Km, Ofx, Eto, Cm, PAS, Cs)       2. Susceptibility test Not performed    2. Negative 2. Culture Not performed | | | Pr. TB database  Sc. TB03/02 journals | |
| 1. DOT regimen modality:    1. Facility based (only)    2. Facility and visiting nurses (mixed)    3. Visiting nurses (only)    4. Mobile DOT (administered in the vehicle)    5. Video Observed DOT (go to q. 10)    6. Home based DOT (go to q. 10) | | | TB01 form | |
| 1. DOT regimen frequency:    1. Every day (except Sunday)    2. Three times a week    3. Two times a week    4. Once a week | | | TB01 form | |
| 1. DOT location    1. TB unit    2. Rural ambulatory | | | TB01 form | |
| 1. Date of DOT interruption    1. _____________________ (dd/mm/yy)    2. No interruption (go to q. 14) | | | TB01 form | |
| 1. Reason of Interruption    1. Patient absent    2. Side effects    3. Comorbidities    4. Prescribed break    5. Drug supply interruption    6. Substance abuse    7. Other reason ____________________ | | | TB01 form | |
| 1. Data of DOT renewal    1. _____ / ____ / ____ (dd/mm/yy)    2. No renewal | | | TB01 form | |
| 1. Date of referral to other TB institution    1. _____ / ____ / ____ (dd/mm/yy)    2. No referral (go to q. 16) | | | TB01 form | |
| 1. Reason of referral    1. DOT continuation    2. Hospitalization    3. Other ______________________ | | | TB03/02 journals | |
| 1. Comorbidities    1. HIV, date of first known diagnosis: ___ / ___ / ___ (dd/mm/yy)    2. HCV, date of first known diagnosis: ___ / ___ / ___ (dd/mm/yy)    3. Diabetes, date of first known diagnosis: __/ ___ / __ (dd/mm/yy)    4. Cerebrovascular conditions, date of first known diagnosis: ___ / ___ / ___ (dd/mm/yy)    5. Other | | | Patient record  TB01 form (for DR-TB)  TB database (for HIV) | |
| 1. Hospitalization due to aggravation of any comorbid condition during TB outpatient treatment period    1. Yes, date of hospitalization ___ / ___ / ___ (dd/mm/yy)    2. No | | | Patient record | |
| 1. Adherence to treatment | | | TB01 form | |
| - 1. At 3 months | Yes | No |  | |
| - 1. At 4 months | Yes | No |  | |
| - 1. At 5 months | Yes | No |  | |
| - 1. At 6 months | Yes | No |  | |
| - 1. At 9 months | Yes | No |  | |
| - 1. At 12 months | Yes | No |  | |
| - 1. At 15 months | Yes | No |  | |
| - 1. At 18 months | Yes | No |  | |
| - 1. At 21 months | Yes | No |  | |
| - 1. At 24 month | Yes | No |  | |
| 1. Date of treatment outcome ____ / ____ / ____ (dd/mm/yy) | | | Pr. TB database  Sc.TB03/02 journals | |
| 1. Treatment outcome    1. Cured    2. Completed    3. Failure (go to 21)    4. Lost to follow-up    5. Died    6. Not evaluated | | | Pr. TB database  Sc.TB03/02 journals | |
| 1. Transferred to    1. DS-TB treatment regime    2. DR-TB treatment regime    3. Other | | | Pr. TB database  Sc.TB03/02 journals | |

**Facility Trial form - (FT)**

*Administered by research assistant, data collection from the document review*

| TB unit ID ____________________ | | |  |
| --- | --- | --- | --- |
| Month / Year | Number of presumptive cases | Number of diagnosed cases | Data source |
| 1. ______ / ______ |  |  | Presumptive TB registration journal |
| 1. ______ / ______ |  |  |  |
| 1. ______ / ______ |  |  |  |
| 1. ______ / ______ |  |  |  |
| 1. ______ / ______ |  |  |  |
| 1. ______ / ______ |  |  |  |
| 1. ______ / ______ |  |  |  |
| 1. ______ / ______ |  |  |  |
| 1. ______ / ______ |  |  |  |
| 1. ______ / ______ |  |  |  |
| 1. ______ / ______ |  |  |  |
| 1. ______ / ______ |  |  |  |
| 1. ______ / ______ |  |  |  |
| 1. ______ / ______ |  |  |  |
| 1. ______ / ______ |  |  |  |
| 1. ______ / ______ |  |  |  |
| 1. ______ / ______ |  |  |  |
| 1. ______ / ______ |  |  |  |
| 1. ______ / ______ |  |  |  |
| 1. ______ / ______ |  |  |  |
| 1. ______ / ______ |  |  |  |
| 1. ______ / ______ |  |  |  |
| 1. ______ / ______ |  |  |  |
| 1. ______ / ______ |  |  |  |
