# Supplementary material for: Addressing challenges in tuberculosis adherence via performance-based payments for integrated case management: protocol for a cluster randomized controlled trial in Georgia
Source: Trials. 2019 Aug 28;20:536. doi: 10.1186/s13063-019-3621-z (PMC6714082; doi:10.1186/s13063-019-3621-z)
Supplement: Supplementary file 5 — Steering committee and data management team. (DOCX 14 kb) [file 13063_2019_3621_MOESM5_ESM.docx]

### Steering committee

A steering committee will oversee trial management and implementation; this committee includes:

1. Dr Tamar Gabunia, Deputy chair of the Country Coordinating Mechanism (not involved in the study) - **Chair**
2. Dr Akaki Zoidze, Principal Investigator, Curatio International Foundation
3. Professor Sophie Witter, PhD, Co-Investigator, Queen Margaret University, Edinburgh
4. Professor Anna Vassal, PhD, Co-Investigator, London School of Hygiene and Tropical Medicine
5. Professor Bruno Marchal, PhD, Co-Investigator, Institute of Tropical Medicine, Antwerp
6. Dr Predrag Duric, PhD, Co-Investigator, Queen Margaret University, Edinburgh
7. Professor Jean Macq, PhD, Faculty of Public Health, Université Catholique de Louvain (not involved in the study)
8. Dr Katherine Fielding, PhD, Department of Infectious Disease Epidemiology, London School of Hygiene and Tropical Medicine (not involved in the study)
9. Dr Nino Berdzuli, Deputy Minister of Labour, Health and Social Affairs of Georgia (not involved in the study)
10. Dr Mamuka Djibuti, PhD, Director, Partnership for Research and Action for Health, Georgia (not involved in the study)
11. Dr. Tsira Chakaia, CSO – Center of Bioethics Studies and Culture, Member of TB Georgian Coalition (not involved in the study)

### Data management team

The data management team for the trial includes:

Ivdity Chikovani (CIF) –Oversee overall data management process, coordinate data collection procedures, oversee data quality control, integration of data from different sources

Maia Uchaneishvili (CIF) - Ensure the security of data and access control;

Lela Sulaberidze – Researcher (CIF) - Data collection according to the data management policy (interviews, observations)

Natia Shengelia – Researcher (CIF) - Data collection according to the data management policy (interviews, observations)

Research assistant (CIF) – Data collection according to the data management policy (documents review- TB registries, patient records, etc.)

Database manager (Consultant) – Data collection according to the data management policy (Data extraction from the national TB database)

TB doctors - will be also involved in the data collection such as patient eligibility screening, enrolment and socio-economic data collection.
