# Supplementary material for: Addressing challenges in tuberculosis adherence via performance-based payments for integrated case management: protocol for a cluster randomized controlled trial in Georgia
Source: Trials. 2019 Aug 28;20:536. doi: 10.1186/s13063-019-3621-z (PMC6714082; doi:10.1186/s13063-019-3621-z)
Supplement: Supplementary file 6 — Trial registration in World Health Organization Trial Dataset. (DOCX 25 kb) [file 13063_2019_3621_MOESM6_ESM.docx]

**World Health Organization Trial Dataset**

1. **Primary Registry and Trial Identifying Number**
   [**ISRCTN14667607**](http://www.isrctn.com/ISRCTN14667607)**.**
2. **Date of Registration in Primary Registry**
   14/01/2019.
3. **Secondary Identifying Numbers**
   NA
4. **Source(s) of Monetary or Material Support**
   Joint Health Systems Research Initiative of the Department of International Development, the Economic and Social Research Council, the Medical Research Council and the Wellcome Trust (March 2017 – February 2021) are funding the research components and intervention training.

The Global Fund to fight HIV/AIDS, TB and Malaria will support the Results Based Financing scheme for 10 intervention sites during the 24 months trial (May 2019-May 2021) through the Tuberculosis Grant.

1. **Primary Sponsor**
   Medical Research Council – UK.
2. **Secondary Sponsor(s)**
   NA.
3. **Contact for Public Queries**
   Maia Uchaneishvili

3 Kavsadze str
Tbilisi
0179
Georgia
+995 (0)322251344
[m.uchaneishvili@curatio.com](mailto:m.uchaneishvili@curatio.com)

1. **Contact for Scientific Queries**
   **Principal Investigator:** Professor Sophie Witter,

[switter@qmu.ac.uk](mailto:switter@qmu.ac.uk), T: +44 (0) 131 474 0000

Institute for Global Health and Development, Queen Margaret University, Edinburgh

Musselburgh, EH21 6UU, Scotland

**Trial contacts:**

Dr. Karin Diaconu

[kdiaconu@qmu.ac.uk](mailto:kdiaconu@qmu.ac.uk), T: +44 (0) 131 474 0000

Institute for Global Health and Development, Queen Margaret University, Edinburgh

Musselburgh, EH21 6UU, Scotland

Dr. Ivdity Chikovani

3 Kavsadze str

Tbilisi

0179

Georgia

+995 (0)322251344

[i.chikovani@curatio.com](mailto:i.chikovani@curatio.com)

1. **Public Title**
   Impact of integrated care and performance-based financing on treatment adherence among pulmonary tuberculosis patients in Georgia
2. **Scientific Title**
   Cluster randomized trial of an integrated care and results-based financing intervention to improve adherence among patients with drug-susceptible and drug-resistant tuberculosis in Georgia
3. **Countries of Recruitment**
   Georgia
4. **Health Condition(s) or Problem(s) Studied**
   Drug susceptible and drug-resistant pulmonary tuberculosis
5. **Intervention(s)**
   Intervention arm: Health providers within intervention sites will form a case management team and be trained in the delivery of integrated tuberculosis care; performance related payments based on monthly records of patients adhering to treatment and quality of care assessments will be disbursed to health providers in these facilities.

Control arm: Standard care.

1. **Key Inclusion and Exclusion Criteria**
   To be eligible for the trial, health facilities must comply with all the following criteria at randomization:

- Facilities must include a TB unit (TB unit at specialised TB care facility, or TB unit integrated into primary healthcare);
- Facilities must have registered at least 20 patients with pulmonary DS-TB as treated in the baseline year used for sampling purposes (2016);
- Facilities must have a pulmonary DS-TB treatment success rate of 82% or less in the baseline year;
- Facilities must have at least one TB doctor and one TB nurse available within the facility;
- Managers of health care facility networks, single private healthcare facility providing general outpatient care, or managers of specialised facilities must consent and agree to participate in the trial, including agreement to disburse performance related payments as set out in the intervention.

Clusters will be excluded if they are taking part in any other clinical trials on treatment regimen, new drugs, adherence to treatment, and/or if outcome monitoring frameworks of the facility are assessed as weak (and therefore inflexible for intervention) by the National TB Directory

To be eligible for participation in the trial, patients must be:

- Aged 18 or over;
- A new or previously treated patients diagnosed bacteriologically or clinically with pulmonary TB (PTB). Diagnosis must have been established by a direct sputum smear microscopy, culture or Gene Xpert MTB/RIF (bacteriologically confirmed case), or X-ray, histological or morphological changes (clinically diagnosed case).
- Assigned to outpatient TB treatment not more than one month prior to enrolment in the study. This restriction (not more than one-month treatment) does not refer to patients whose most recent treatment outcome was failure and who were assigned to a new treatment regimen;
- Assigned to outpatient TB treatment at outpatient facility or at home (through DOT or video observed therapy (VOT)) according to the national TB treatment guidelines.

Patients will be excluded if:

- Diagnosis is extra-pulmonary TB;
- TB outpatient treatment was started more than one month prior to enrolment in the study;
- Patients with DS-TB have undergone more than two months of hospitalisation or patients with DR-TB have undergone more than six months of hospitalisation;
- Patients are known at the start of treatment to require the treatment longer than it is recommended by the Georgian TB Management Guidelines for the appropriate type of TB (Appendix 2);
- Patients are custody patients;
- Patients are involved in other clinical studies related to TB treatment;
- Patients are leaving the area within next six months

1. **Study type**
   This is a pragmatic, cluster randomised, controlled, superiority trial with two parallel groups, fixed number of clusters of unequal size and a primary outcome of loss to follow-up among drug-sensitive and drug-resistant TB outpatients. A clustered design – featuring health facilities as clusters – was chosen as facilities represent the ideal frame to disburse performance related payments to TB providers. Randomization will be constrained and stratified according to facility operation type (i.e. integrated vs. specialized care management) and clusters will be allocated to intervention and control arms on a 1:1 ratio. Via constrained randomization we will assure no substantive baseline imbalance exists among clusters in relation to patient load (values from 2016 and 2017), treatment related loss to follow-up and success among drug-susceptible patients (values from 2016), number of human resources (doctors and nurses) and their average salary***.***
2. **Date of First Enrollment**
   24 May 2019
3. **Sample Size**
   The required sample size to detect a 6% reduction in loss to follow-up among DS-TB patients and a 20% reduction in loss to follow-up among DR-TB patients is 948 and 136 patients respectively.
4. **Recruitment Status**
   Recruiting
5. **Primary Outcome(s)**
   The primary outcome is loss to follow-up among adult drug-sensitive tuberculosis patients. Loss to follow-up is defined as the difference between the two intervention arms in the proportion of patients who did not start treatment after diagnosis or whose treatment was interrupted for 2 consecutive months or more. The outcome will be assessed based on patient collected data at every treatment month.

20. **Key Secondary Outcomes**
The following secondary outcomes will be studied:

1. loss to follow-up among drug-resistant tuberculosis patients (similarly defined as for drug-sensitive patients) at each month of a treatment;

2. adherence to treatment among drug-sensitive tuberculosis patients every month of treatment.

Definition:

- from the start of the treatment up to treatment completion, not more than one missing day per month if patient visits a facility 8-12 times during a month to fulfil DOT (DOT is performed 2 or 3 days per week. Every other day the patient receives drugs at home)
- from the start of the treatment up to treatment completion, no single missing day per month if patient visits a facility 4 times during a month to fulfil DOT (some patients visit a facility only once a week for DOT and take drugs for home-taking)

3. adherence to treatment among drug-resistant tuberculosis patients.

Definition: Adherence to DOT for patients with pulmonary DR-TB is defined as not more than three missing days per month (DOT is performed 26 days per month - 6 days a week except Sunday) from the start of the treatment.

4. treatment success among drug-sensitive tuberculosis patients.

Treatment success is defined as per WHO (21). The treatment success rate will be calculated as the difference in the proportion of patients classed as successfully treated between trial arms at 6, 12 and 24 months.

1. **Ethics Review**
   Ethical approvals for this research have been secured from the National Center for Disease Control and Public Health of Georgia (IRB # 2018-01) and Queen Margaret University, Edinburgh, UK (IRB # REP0172).
2. **Completion date**
   1 May 2021
3. **Summary Results**
   Not applicable.
4. **IPD sharing statement**
   The datasets generated during and/or analysed during the current study are/will be available upon request from the Co-investigator Dr. Ivdity Chikovani email: i.chikovani@curatio.com . Clinical trial data including patient characteristics and outcomes will be available 2 years post trial completion to researchers or practitioners in the field, upon reasonable request. Data may be used for confirmatory analyses and meta-analyses by encrypted file sharing. Consent was obtained from participants and only completely anonymized datasets will be available.
